# Comprehensive analysis of the clinical and biological significances of cholesterol metabolism in lower-grade gliomas

**DOI:** 10.1186/s12885-023-10897-0

**Published:** 2023-07-24

**Authors:** Rui Tao, Ruoyu Huang, Jingchen Yang, Jiangfei Wang, Kuanyu Wang

**Affiliations:** 1 Department of Neurosurgery, Beijing Tiantan Hospital, Capital Medical University, No.119 South 4th Ring West Road, Fengtai District, Beijing, 100070 China; 2 Department of stereotactic radiosurgery, Beijing Tiantan Hospital, Capital Medical University, No.119 South 4th Ring West Road, Fengtai District, Beijing, 100070 China; 3 Department of Molecular Neuropathology, Beijing Neurosurgical Institute, Capital Medical University, Beijing, 100070 China

**Keywords:** Lower-grade gliomas, Risk signature, Tumor immune microenvironment, Cholesterol metabolism, Prognosis

## Abstract

**Background:**

As a component of membrane lipids and the precursor of oxysterols and steroid hormones, reprogrammed cholesterol metabolism contributes to the initiation and progression of multiple cancers. Thus, we aim to further investigate the significances of cholesterol metabolism in lower-grade gliomas (LGGs).

**Methods:**

The present study included 413 LGG samples from TCGA RNA-seq dataset (training cohort) and 172 LGG samples from CGGA RNA-seq dataset (validation cohort). The cholesterol metabolism-related signature was identified by the LASSO regression model. Bioinformatics analyses were performed to explore the functional roles of this signature in LGGs. Kaplan-Meier and Cox regression analyses were enrolled to estimate prognostic value of the risk signature.

**Results:**

Our findings suggested that cholesterol metabolism was tightly associated clinicopathologic features and genomic alterations of LGGs. Bioinformatics analyses revealed that cholesterol metabolism played a key role in immunosuppression of LGGs, mainly by promoting macrophages polarization and T cell exhaustion. Kaplan-Meier curve and Cox regression analysis showed that cholesterol metabolism was an independent prognostic indicator for LGG patients. To improve the clinical application value of the risk signature, we also constructed a nomogram model to predict the 1-, 3- and 5-year survival of LGG patients.

**Conclusion:**

The cholesterol metabolism was powerful prognostic indicator and could serve as a promising target to enhance personalized treatment of LGGs.

**Supplementary Information:**

The online version contains supplementary material available at 10.1186/s12885-023-10897-0.

## Introduction

As common and malignant primary brain tumors in adults, lower-grade gliomas(LGGs) are classified as histologic CNS WHO grade 2 or 3 in the World Health Organization (WHO) classification [1–3]. LGGs include astrocytoma and oligodendroglioma in terms of histological classification. It also contains gliomas featured IDH-wildtype and IDH-mutated. Although, LGGs have lower incidence rate and malignancy compared with glioblastoma (GBM), IDH-wildtype(CNS WHO grade 4), it also causes considerable mortality and presents a therapeutic challenge due to the heterogeneity of their clinicopathological features [4, 5]. Besides, prognosis of LGGs patients is variable, and overall survival (OS) time also ranges widely (from 1 year to 15 years) [6]. Despite intensive treatment with surgical resection, radiotherapy, and chemotherapy, tumor relapse is inevi [7]. Thus, to find more effective treatments for LGGs, it is vital to better understand the mechanism of initiation and progression of this disease.

Cholesterol is a key component of membrane lipids of cells and organelles, which is essential for the survival of cells [8]. Recently, several studies have proved that reprogrammed cholesterol metabolism contributes to the malignant progression in a variety of human cancers [9, 10]. For example, high concentration of cholesterol can promote the migration and invasion of cancer cells in renal carcinoma and melanoma [11, 12]. Besides, as a precursor of steroid hormones and bile acids, cholesterol also participate the initiation and progression of colon, breast, and prostate cancer [13–15]. Consequently, the blockade of cholesterol metabolism or cholesterol depletion is considered to be a promising treatment strategy of cancers and arouse wide attention [16]. More interestingly, cholesterol tend to enrich in tumor microenvironment (TME) and have significant impact on the immune and inflammatory responses [17, 18]. Relevant studies have reported that cholesterol in TME could induce the overexpression of immune checkpoints (such as *PD-1*, *2B4*, *LAG-3* and *TIM-3*) and functional exhaustion of tumor-infiltrating lymphocytes (TILs), which eventually lead to immune evasion of cancer cells [19]. These findings suggested that cholesterol metabolism may serve as an effective target to improve the antitumor efficacy of immunotherapy. Thus, the comprehensive analysis of cholesterol metabolism could provide important reference for individual treatment of LGG patients.

## Materials and methods

### Data collection

413 LGG samples from The Cancer Genome Atlas (TCGA) database were selected in this study as discovery cohort. Among them, 149 were astrocytoma, 108 oligoastrocytoma, and 156 oligodendroglioma. 335 patients were diagnosed as IDH mutated gliomas while 76 wildtype. 1p/19q-codeleted were detected in 138 patients while 275 not (supplementary Table 2). The clinical information and RNA sequencing data were downloaded from public access website (https://portal.gdc.cancer.gov/projects/TCGA-LGG). Meanwhile, a total of 172 LGG samples with RNA sequencing data and follow-up information were obtained from the Chinese Glioma Genome Atlas (CGGA) database (http://www.cgga.org.cn/download.jsp) for validation cohort. Among them, 66 were astrocytoma, 67 oligoastrocytoma, and 39 oligodendroglioma. 127 patients were diagnosed as IDH mutated gliomas while 45 wildtype. 1p/19q-codeleted were detected in 34 patients while 110 not (supplementary Table 3). In our previous studies, we have elaborated the details of construction and management of the CGGA database [20, 21]. All patients enrolled in this study signed informed consent. This study was approved by the Institutional Review Board (IRB) of Tiantan Hospital and kept consistent the Declaration of Helsinki. Besides, specific tumor anatomic structure with RNA sequencing data from Ivy Glioblastoma Atlas Project (http://glioblastoma.alleninstitute.org/static/download.html) were also enrolled in this study to investigate the heterogeneity of cholesterol metabolism in glioma [22]. All the RNA sequencing data were log2-transformed before analysis. The test of normality has been performed in our pervious study [23].

### Identification of cholesterol metabolism-related clusters

Based on the expression of 84 cholesterol metabolism-related genes (supplementary Table 1), we performed unsupervised clustering using the R package “ConsensusClusterPlus” to identify robust clusters of LGG patients in TCGA cohort [24]. In the consensus clustering analysis, the value range of evaluated K was from 2 to 10. The optimal K was identified by cumulative distribution function (CDF) and consensus matrices. Besides, same analysis was also performed in CGGA cohort to evaluate the reliability and reproducibility of the acquired clusters.

### Development of the risk signature

As a helpful tool for high-dimensional regression analysis, the LASSO Cox regression model was adopted for construction of the optimal risk signature with R package “glmnet” [25, 26]. Firstly, in TCGA cohort, cholesterol metabolism-related gene list was enrolled in this LASSO Cox regression model. The genes and their coefficients were selected by the best penalty parameter λ and the smallest 10-fold cross validation. Subsequently, the risk score of each LGG patient was calculated based on the gene expression level and regression coefficient (Coeffs). The formula was listed as followed: $Signature risk score={\sum }_{i=1}^{n}\beta ixi$. $\beta i$ and $xi$ indicate the regression coefficients and relative expression of each gene enrolled in the risk signature, respectively. In CGGA cohort, the risk score of each case was also calculated with genes expression and the same regression coefficients for validation. The risk scores of patients were ranked from highest to lowest. The top 50% was defined as high risk group while the bottom 50% low risk group. High risk meant higher occurrence of end-point events which represented to the death of patients caused by glioma.

### Cell culture and transfection

Human glioma cell line U87 and LN229, which were purchased from the Institute of Biochemistry and Cell Biology, Chinese Academy of Sciences were used in this study. All glioma cell lines were cultured in DMEM medium (Gibco; Thermo Fisher Scientific, United States) supplemented with 10% fetal bovine serum (FBS, Gibco; Thermo Fisher Scientific) and maintained in an incubator (37 °C, 5% CO2). The *SOAT1* small interference RNA (siRNA) and negative control (NC) were synthesized by RiboBio Co., Ltd. (Guangzhou, China). U87 and LN229 cell lines was transfected with siRNA or NC (50 nM) using the transfection reagent (Polyplus-transfection Co., Ltd., France) for 48 h. Then, fresh medium without siRNA or NC was added to the cells. The sequences for *SOAT1* siRNA are as follows: siRNA1 50-TCAGCACACTTGTAGTAGA-30; siRNA2 50-GTGACAGGATGTTCTATAA-30; siRNA3 50-GACTTGTCGTTACGTGTTT-30. In this study, all glioma cell lines have been authenticated using STR profiling.

### Quantitative PCR and western blot

The SYBR SuperMix kit (Bio-Rad Laboratories, Inc.) and the 7,500 Fast Real-Time PCR system (Applied Biosystems, United States) were used to perform quantitative PCR (qPCR). The relative mRNA expression levels of *SOAT1* were normalized to *β-ACTIN* and were calculated using the 2^‑ΔΔCT^ method. The primer sequences of *SOAT1* and *β-ACTIN* were synthesized by GENEWIZ Co. (Beijing, China). The primer sequences were as follows: *SOAT1*, forward 50-GAAGTTGGCAGTCACTTTGATGA-30, and reverse 50- GAGCGCACCCACCATTATCTA-30 and *β-ACTIN*, forward 50- CACCATTGGCAATGAGCGGTTC-30, and reverse 50- AGGTCTTTGCGGATGTCCACGT-30. Western blot (WB) analysis was performed with rabbit anti-*SOAT1* polyclonal antibody (1:800, Santa-cruz) and mouse anti-*β-ACTIN* antibody (1:5,000, Abcam). Goat anti-rabbit/anti-mouse IgG-HRP (1:5,000, ZSGB-Bio, China) was used as secondary antibodies. In order to reduce the probable miscellaneous bands, we cropped the membrane and incubated it with the primary antibody of β-actin and SOAT1 separately.

### Cell Migration and cell proliferation assays

The cell migration and cell proliferation assays were performed as previously reported [27].

### Immunohistochemistry

In this study, we performed immunohistochemical (IHC) staining of *SOAT1*, *IBA1* and *CD163* with specific primary antibodies (anti-*SOAT1*, 1:500 dilutions, Santa cruz, USA, anti-*IBA1*, 1:500 dilutions; anti-*CD163*, 1:500 dilutions; Abcam, Cambridge, United Kingdom). The IHC staining were performed as previously reported [27].

### Bioinformatic analysis

In this study, principal components analysis (PCA) was applied to explore the differences within stratified patients with R package “princomp” [28]. Somatic mutations and somatic copy number alternations (CNAs) data were downloaded from TCGA database. The association between CNAs and risk signature was assessed by GISTIC2.0 analysis [29]. Pearson correlation analysis was employed to identified the genes tightly correlated with risk score (Pearson ∣R∣> 0.4). Subsequently, the functional roles of these genes were annotated by Gene ontology (GO) analysis and Kyoto Encyclopedia of Genes and Genomes pathway enrichment (KEGG) analysis on DAVID website (http://david.abcc.ncifcrf.gov/home.jsp) [30]. Metascape was employed to further validated the results of GO and KEGG analysis (http://metascape.org/). Gene-set enrichment analysis (GSEA) was performed to detect the differences of biological functions between samples stratified by risk score [31]. Receiver operating characteristic (ROC) curve analysis was carried out to assess the accuracy of risk signature in predicting the 1-, 3- and 5-year survival rate with R package “pROC” [32]. ESTIMATE algorithm was applied to calculate the abundance of stromal and immune cells with R package “estimate”, and tumor purity of each sample was calculated according to the formula proposed by Yoshihara et al [33]. Furthermore, CIBERSORT algorithm and xCell algorithm were also enrolled in this study to estimate the infiltration of immune cells in LGG samples (https://cibersort.stanford.edu/, https://xcell.ucsf.edu/)[34, 35]. Gene set variation analysis (GSVA) analysis was performed to detect the association between the risk signature and T-cell-mediated immune response in LGG samples [36]. R language v3.4.3 was the primary tool for bioinformatic analysis in this study.

### Statistical analysis

Student’s t test, one-way ANOVA and Chi-square test were carried out to compare differences among groups. Kaplan-Meier (K-M) survival analyses with log-rank tests were employed to describe the survival distribution. While, univariate and multivariate Cox regression analyses were conducted to identify the factors with independent prognostic value. Ideally, the number of end-point event should be at least 10 times the number of independent variables in COX regression analysis. Selecting too many variables could lead to the inaccuracy of the results [37–39]. In this study, the end-point events were 77 in TCGA and 50 in CGGA cohort. So we chose no more than 7 variables linking closely to the prognosis of gliomas to proceed the univariate Cox regression analysis. IDH mutation status, 1p19q co-deletion status, MGMT promoter methylation status, age at diagnosis, WHO grade, gender, and risk score were included in univariate analysis in both TCGA and CGGA database. Forward stepwise selection process was further used to decide which variables were included in multivariate analysis. The entry criteria was set as α = 0.05.The statistical analysis were conducted using R language v3.4.3 and GraphPad Prism 7.0 (GraphPad Inc., San Diego, CA, SA) in this study. P < 0.05 was considered statistically significant.

## Results

### Cholesterol metabolism-related clusters were identified in LGGs

**Fig. 1:**
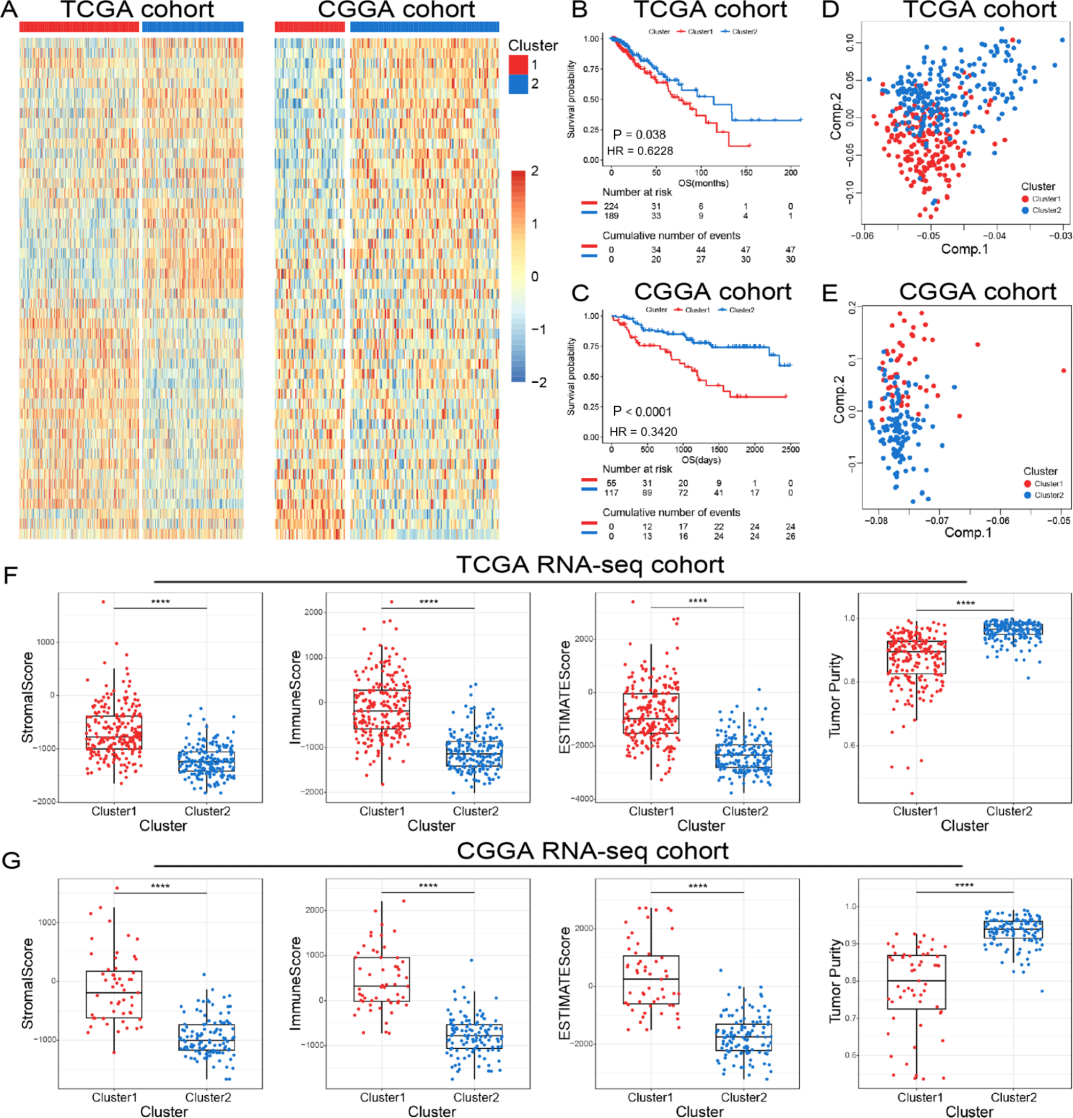
Consensus clustering identified two cholesterol metabolism-related clusters in TCGA and CGGA cohorts. **(A)** Heatmap of two clusters identified by the cholesterol metabolism-related genes. The x axis referred to tumor samples of different patients with glioma. The y axis represented top 50 cholesterol metabolism related genes which had higher median absolute deviation (MAD) value among different tumor samples. Higher MAD value favored cluster analysis. **(B, C)** K-M survival analyses of patients in two clusters. **(D, E)** Principal components analysis of two clusters. **(F, G)** Comparison of StromalScore, ImmuneScore, ESTIMATEScore, and tumor purity between two clusters. **** P < 0.0001

In order to explore the functional role of cholesterol metabolism in the initiation and progression of LGGs, consensus clustering analysis was performed with RNA sequencing data from TCGA and CGGA databases. Algorithm of consensus clustering analysis was based on relative expression level of top 50 cholesterol metabolism related genes which had higher median absolute deviation (MAD) among LGGs patients enrolled in this study. LGGs patients were divided into two clusters based on this algorithm (Fig. 1A). The optimal number of subgroups was assessing by CDF curves and consensus matrices (supplementary Fig. 1). To further decipher the differences in clinicopathological features between these two clusters, Chi-square test was performed on the clinical data from TCGA and CGGA databases. The results suggested that patients in Cluster2 had the characteristics of lower grade, Neural or Proneural subtypes, isocitrate dehydrogenase (*IDH*) mutated, O6-methylguanine-DNA methyltransferase (*MGMT*) promoter methylated, and 1p/19q codeleted. Meanwhile, patients in Cluster1 tended to have opposite clinicopathological features (supplementary Tables 2 and 3). Besides, the K-M survival curve analysis suggested that LGG patients in Cluster2 had significantly better prognosis in comparation with ones in Cluster1 in both TCGA and CGGA cohorts (Fig. 1B C). By applying the principal component analysis (PCA), we found a significant difference in the expression portraits between these two clusters (Fig. 1D and E). Moreover, we also enrolled immune-related tools in this study to further investigate the immune heterogeneity between these two clusters in TCGA and CGGA cohorts. The distribution of stromal and immune content of each cluster were estimated by computing Estimate algorithm [33]. As shown in Fig. 1F and G, the StromalScore, ImmuneScore and ESTIMATEScore of Cluster2 were significantly lower than Cluster1, suggesting that the abundance of stromal and immune cells in samples of Cluster2 was lower. Besides, we also observed a reduction of tumor purity in Cluster1, suggesting that samples in Cluster1 had a higher level of immunocyte infiltration in both CGGA and TCGA cohorts (Fig. 1F and G). The results indicated that the cholesterol metabolism status was tightly correlated with molecular features, immunocyte infiltration and prognosis of LGG patients.

Additionally, we also assessed the significance of three-classification according to the CDF curves and consensus matrices (supplementary Fig. 2A). Although the survival of patients in three clusters was statistically different (supplementary Fig. 2B and 2 C), but PCA analysis revealed no obvious difference (supplementary Fig. 2D and 2E).

### Construction of a cholesterol metabolism-related signature in LGGs

**Fig. 2:**
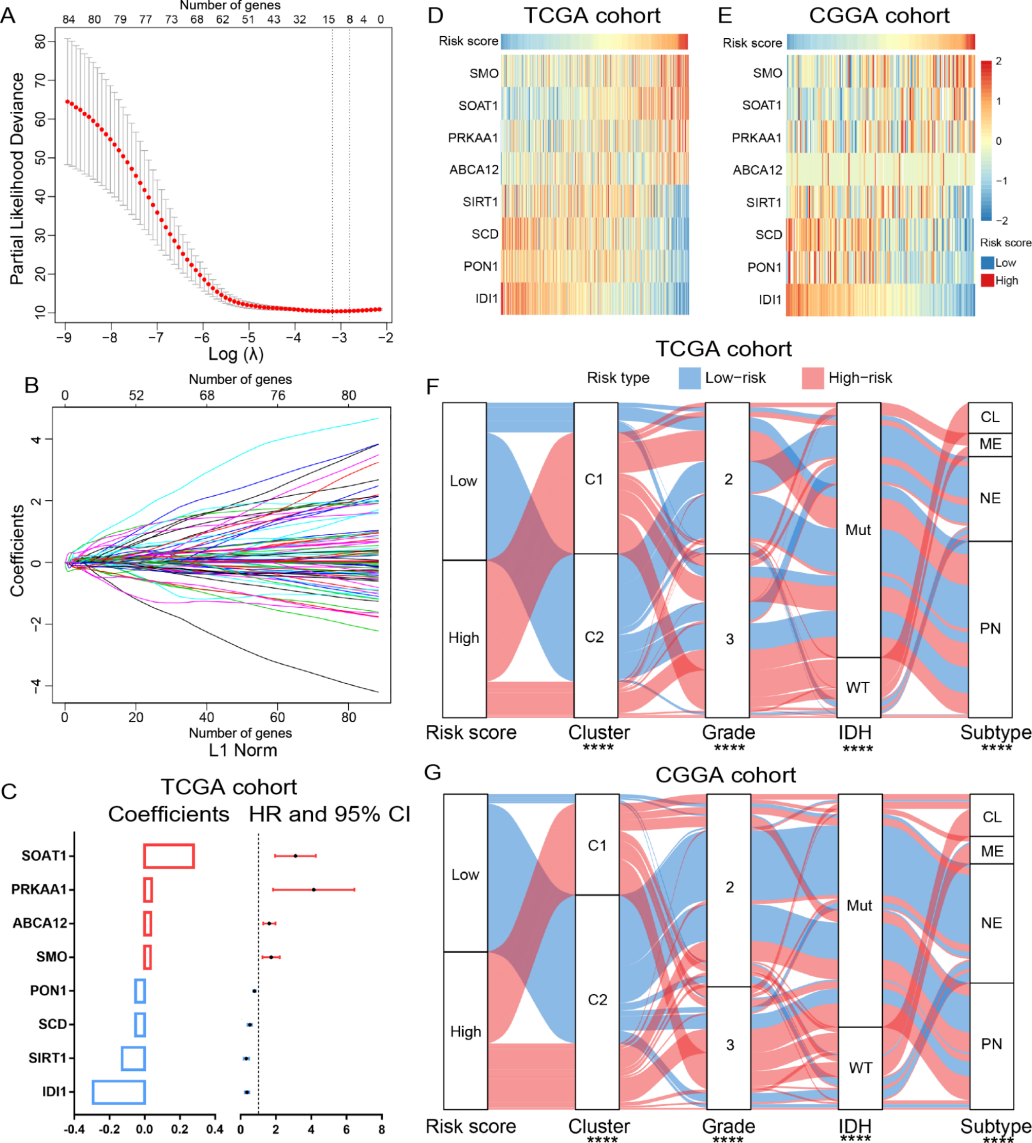
Identification of the cholesterol metabolism-related risk signature. **(A)** Cross-validation for tuning parameter (λ) screening in the LASSO Cox regression model. **(B)** The LASSO coefficient profiles of cholesterol metabolism-related genes. **(C)** The 8 core genes’ coefficients in LASSO Cox regression model and their hazard ratios (HRs) with 95% confidence intervals (CIs) in univariate Cox regression analysis. **(D, E)** Heat maps of 8 core genes in the cholesterol metabolism-related risk signature (F, G) The association among risk score, cluster, WHO grade, IDH mutation status, and molecular subtypes of LGG samples was exhibited by Sankey diagram and examined by Chi-square test. * P < 0.05, ** P < 0.01, *** P < 0.001, **** P < 0.0001

To estimate the cholesterol metabolism status and predict the clinical outcome of LGG patients more accurately, we constructed a cholesterol metabolism-related risk signature in this study. Eight genes (*SMO, SOAT1, PRKAA1, ABCA12, SIRT1, SCD, PON1* and *IDI1*) with non-zero regression coefficients were identified in TCGA cohort (Fig. 2A and B). The core genes with coefficients greater than 0 could serve as risk factors, while the genes with coefficients less than 0 had protective effect (Fig. 2C and supplementary Fig. 3). The heat maps exhibited an overview of correlation between the risk score and the expression of cholesterol metabolism-related genes that constitute the risk signature. (Figure 2D and E). After dividing the patients into two groups evenly according to the risk score, the Sankey diagram showed that the clinicopathological characteristics of cases in the high-risk group were significantly different from those in low-risk group (Fig. 2F and G).

### Association of the risk signature with clinical and molecular features in LGGs

**Fig. 3:**
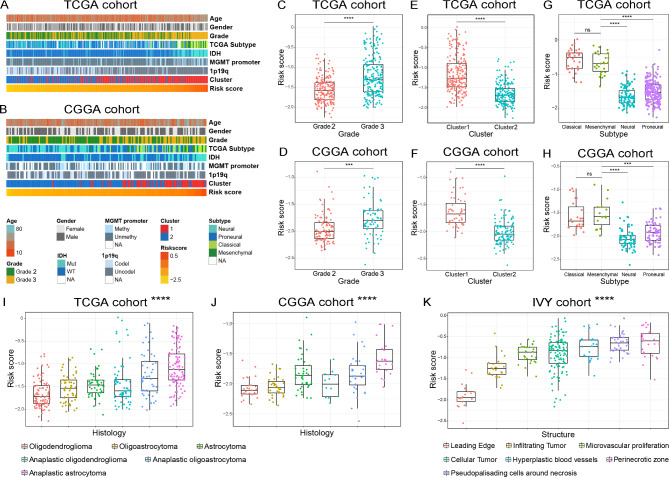
Association between the risk signature and clinicopathological characteristics in TCGA **(A)** and CGGA **(B)** databases. **(C-J)** The association between risk score and WHO grade **(C, D)**, cholesterol metabolism-related clusters **(E, F)**, molecular subtypes **(G, H)** and histopathologic classification **(I, J)** of LGG patients. **(K)** The distribution of risk score in the specific tumor anatomic structure of glioma samples from IVY cohort. *** P < 0.001, **** P < 0.0001, ns: no statistically significant

Due to the characteristic of high histopathological heterogeneity of LGGs, we further investigate the association between the risk signature and clinicopathology features of LGGs in TCGA and CGGA cohorts (Fig. 3A and B). We found that the risk score was positively correlated with the WHO grades of LGGs (Fig. 3C and D) and higher risk score was also enriched in LGG samples in Cluster1 (Fig. 3E F). Additionally, the risk score was significantly increased in samples with the molecular characteristics of *IDH*-wildtype, chromosome 1p/19q non-codeleted and *MGMT* promoter unmethylated, which were generally thought to drive the malignant progression of glioma (supplementary Fig. 4). Moreover, when we detected the distribution of the risk score in four molecular subtypes defined by TCGA network, the results showed that the risk score of Classical and Mesenchymal subtypes were much higher than Neural and Proneural subtypes (Fig. 3G H). Besides, among all histopathologic types of LGGs, anaplastic astrocytoma had the highest risk score (Fig. 3I J). Considering the heterogeneity of gliomas, RNA sequencing data from Ivy Glioblastoma Atlas Project database were also enrolled in this study to investigate the cholesterol metabolic status of specific tumor anatomic structure of gliomas. We found that the tumor tissues from perinecrotic zone, which involved in the drug resistance of gliomas had the highest risk score (Fig. 3K).

**Fig. 4:**
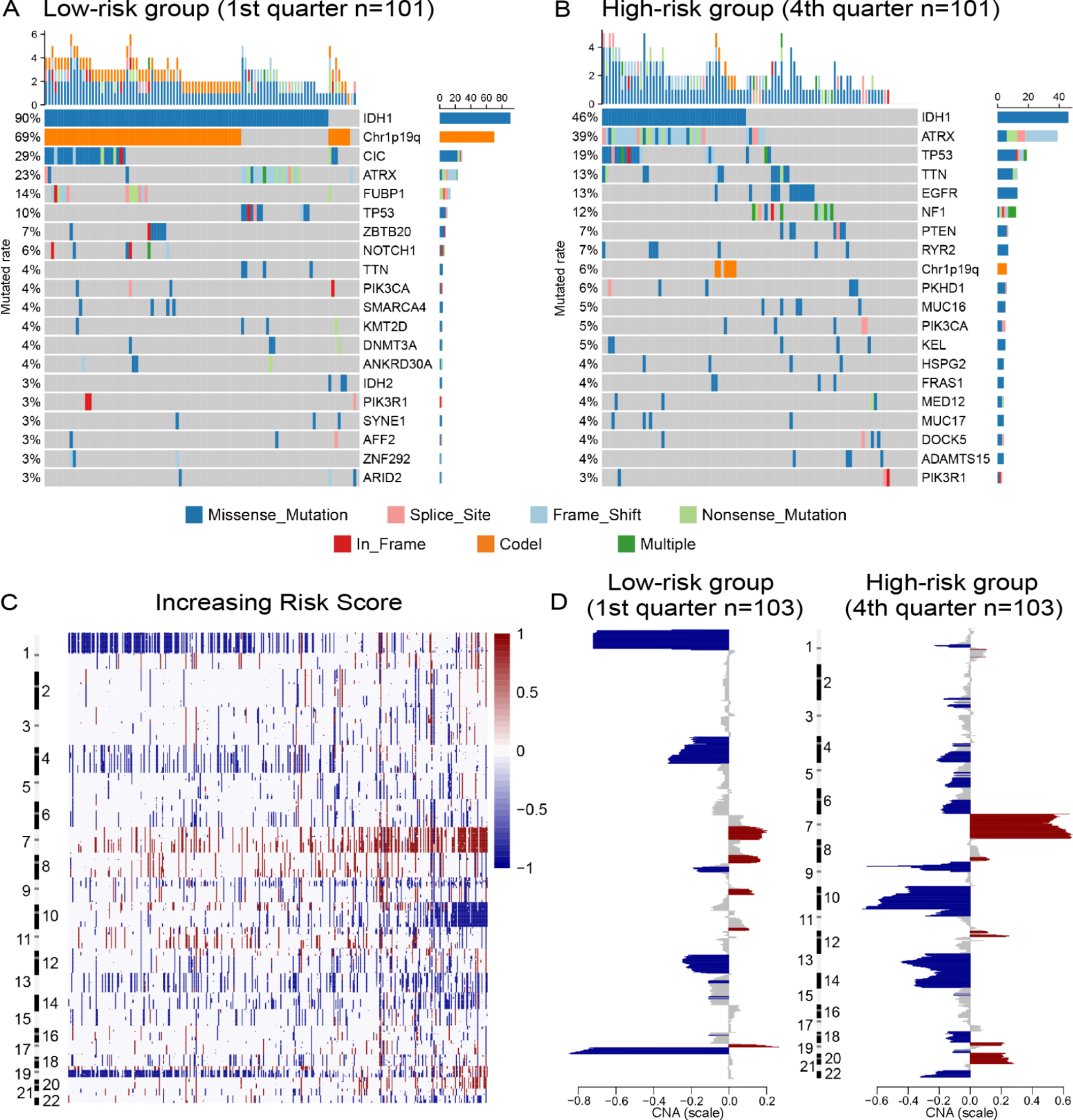
The risk signature associates with distinct patterns of genomic alterations in TCGA cohort. **(A, B)** Comparison of somatic mutations between LGG samples in low-risk group (1st quarter) and high-risk group (4th quarter). **(C)** The overall CNAs profiles with increasing order of the risk score. **(D)** Comparison of CNAs spectrum between LGG samples in low-risk group (1st quarter) and high-risk group (4th quarter). The amplification (red) and deletion (blue) of chromosome segment are presented

Somatic mutations and CNAs are the characteristic changes in cancer cells, which also drive the initiation and progression of LGGs. Here, we comprehensively analyzed somatic mutation and CNAs data from TCGA database to further explore the association between genomic instability and abnormal cholesterol metabolism in LGG samples. First, we divided all samples into two groups evenly according to the order of increasing risk score. We found that the incidence of 1p/19q codeletion and mutations of *CIC (P < 0.001)*, *FUBP1(P = 0.003)* and *NOTCH1(P = 0.028)* were mainly observed in low-risk group (supplementary Fig. 5). Meanwhile, the mutations of *TTN(P = 0.044)*, *NF1(P = 0.007)*, *EGFR(P = 0.002)* and *PTEN(P = 0.044)* were observed enriched in high-risk group (supplementary Fig. 5). When we classified all samples into four groups according to the risk score, the differences became more obvious in 1st and 4th quarter groups (Fig. 4A and B). Besides, we also found that *IDH1* and *ATRX* mutations, as representative molecular features of gliomas, had a high frequency in each group (Fig. 4A and B, supplementary Fig. 5). As a common driver of tumorigenesis, TP53 mutation was also found significant higher in high-risk group (supplementary Fig. 5, P = 0.021). In addition, the somatic CNAs were also investigated between samples with high and low risk score. GISTIC2.0 analysis revealed that 1p/19q codeletion tended to be enriched in low-risk group (Fig. 4C and D). Whereas, the incidence of Chr 7 amplification accompanied Chr 10 loss was increased along with increasing of risk score (Fig. 4C and D). These findings suggested that cholesterol metabolic status might play an essential role in the initiation and progression of LGGs.

### Biological processes associated with the risk signature

**Fig. 5:**
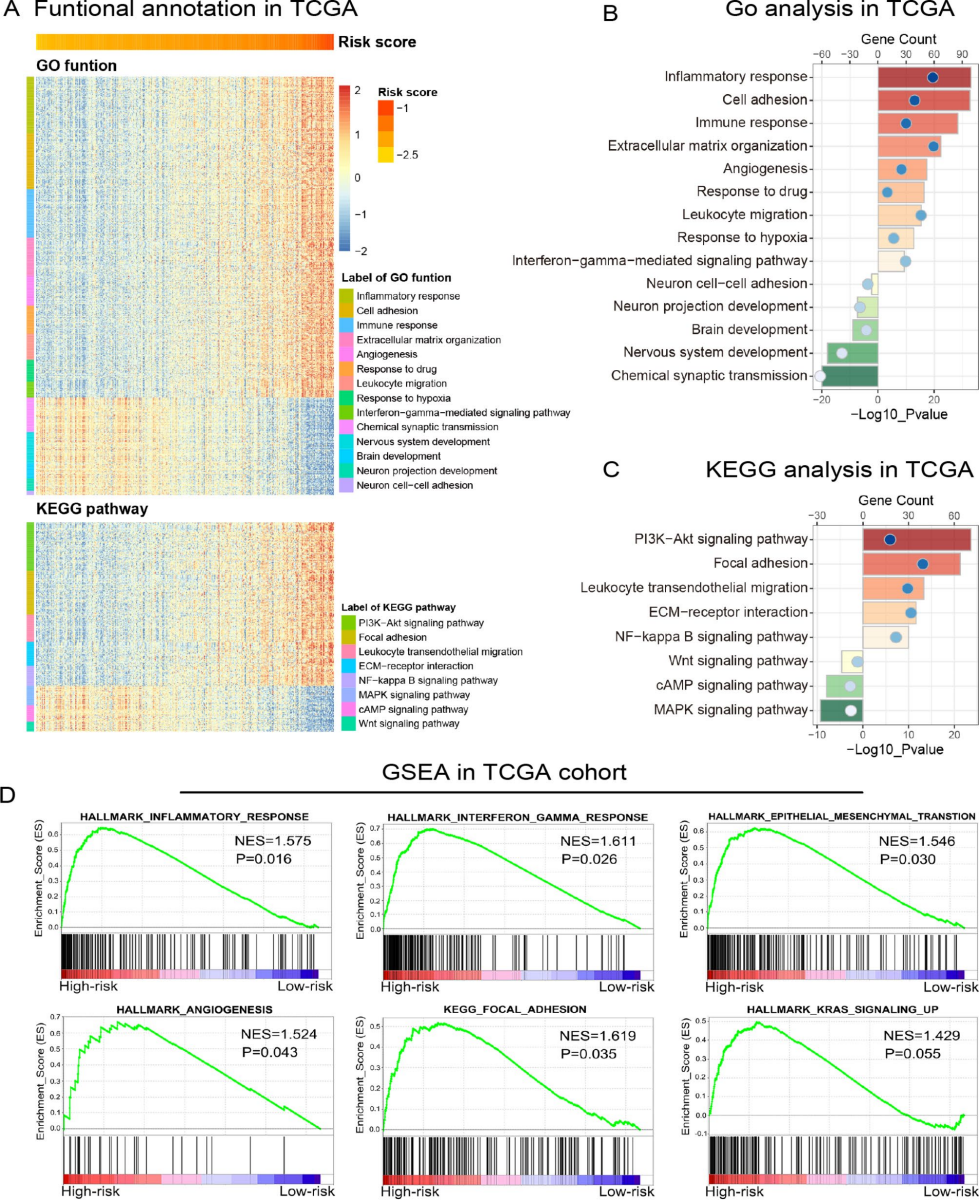
Functional annotation of the risk signature in TCGA cohort. **(A)** The heatmaps showed the association between risk score and cholesterol metabolism-related biological functions and pathways, which were identified by GO and KEGG pathway analyses. **(B, C)** GO and KEGG pathway analyses were performed via the DAVID website to investigate the biological processes tightly correlated to the risk signature. **(D)** GSEA analysis was performed to explore the biological functions that were significantly enriched in LGG samples from high-risk group

To clarify the biological functions tightly correlated to the risk signature in gliomas, GO function analyses were performed in LGG samples of TCGA cohort. The results suggested that the biological processes, such as inflammatory response, immune response, cell division, angiogenesis, response to hypoxia and drug were significantly enriched in the high-risk group. However, normal biological processes including neuron cell-cell adhesion, brain development and nervous system development were negatively correlated with risk score (Fig. 5A and B). In addition, by performing KEGG pathway analysis in TCGA cohort, we found that the risk signature was positively related to Focal adhesion, PI3K-AKT and NF-kappa B signaling pathways and negatively related to WNT, cAMP and MAPK signaling pathways (Fig. 5A C). Furthermore, GSEA also revealed the hallmarks of malignant tumors, including inflammatory response, interferon gamma response, epithelial-mesenchymal transition, angiogenesis, focal adhesion and KRAS signaling, were significantly enriched in LGGs with higher risk score. (Fig. 5D). To ensure the stability and repeatability of these findings, same analyses were also performed in CGGA cohort and consensus results were obtained (supplementary Fig. 6). Metascape was performed to annotate the functional roles of genes which were positively correlated with the risk score (Pearson correlation analysis, R > 0.4), and the results were similar to GO analysis and GSEA in both TCGA and CGGA cohorts (supplementary Fig. 7). These results suggested that cholesterol metabolic status had significant effects on the malignant progression and immunologic microenvironment of LGGs.

### Cholesterol metabolism was relevant to tumor immunologic microenvironment in LGGs

**Fig. 6:**
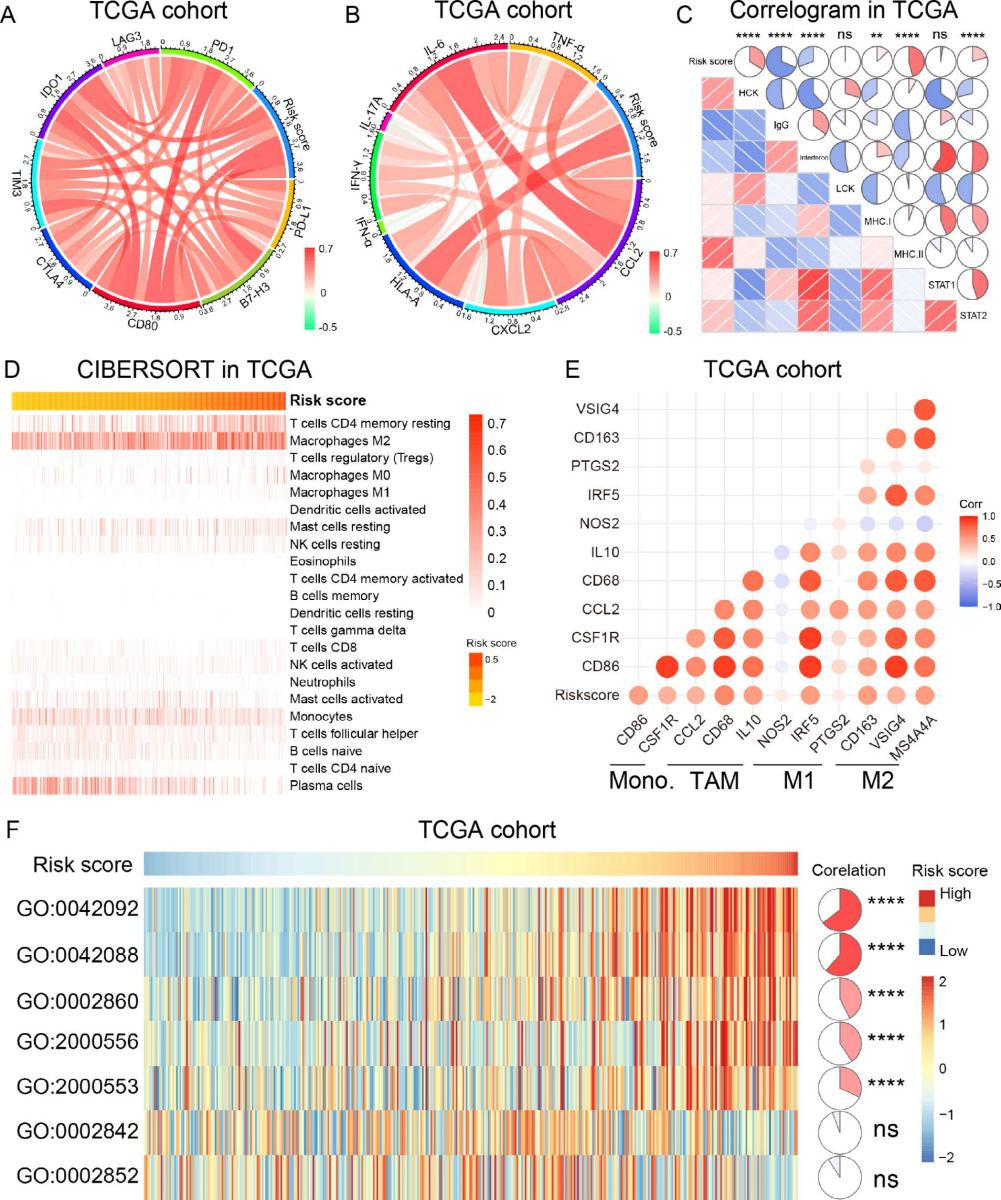
Cholesterol metabolism participated in the regulation of tumor immune microenvironment in TCGA cohort. **(A)** Correlation analysis between the risk signature and immune checkpoints in LGG samples. **(B)** Correlation analysis between the risk signature and inflammatory hallmarks in LGG samples. **(C)** The association between the risk signature and inflammatory metagenes. **(D)** The proportion of immune cells infiltrated into LGG samples was evaluated by CIBERSORT. **(E)** The association between the risk signature and biomarkers of monocytes and macrophages in different phase. **(F)** The relationship between the risk signature and T-cell-related immunity. GO:0042092: T-helper 2 type immune response; GO:0042088: T-helper 1 type immune response; GO:0002860: positive regulation of natural killer cell-mediated cytotoxicity directed against tumor cell target; GO:2,000,556: positive regulation of T-helper 1 cell cytokine production; GO:2,000,553: positive regulation of T-helper 2 cell cytokine production; GO:0002842: positive regulation of T cell-mediated immune response to tumor cell; GO:0002852: regulation of T cell-mediated cytotoxicity directed against tumor cell target. ** P < 0.01, **** P < 0.0001, ns: no statistically significant

As shown in the bioinformatics analysis above, cholesterol metabolism played an essential role in the immune and inflammatory response of LGGs. Thus, to further understand the underlying molecular mechanism, CIRCOS analysis with immune checkpoints and inflammatory biomarkers were enrolled in this study [40]. The risk signature demonstrated a positive correlation with the expression level of immune check points in both TCGA and CGGA cohorts, suggesting a severe immunosuppression in LGG samples of high-risk group (Fig. 6A and supplementary Fig. 8A). Besides, we also found that the risk signature was also positively corelated with most of inflammatory factors (especially *HLA-A*, *IL-6*, *IFN-γ* and *CCL2*) [41, 42]. These results indicated that cholesterol metabolism might participate in the activation of T-cell and macrophage-mediated inflammatory responses in LGGs (Fig. 6B and supplementary Fig. 8B). To verify this, we further analyzed seven metagenes described in previous studies to clarify the role of cholesterol metabolism in the inflammatory response of LGGs [43]. As shown in Fig. 6C and supplementary Fig. 8C, the risk score was positively associated with *HCK*, *MHC-II* and *STAT2*, while negatively associated with IgG in TCGA and CGGA cohorts, indicating that the risk score upregulated in the signaling transduction of T cells and macrophages, but negatively correlated to B lymphocytes activation. The CIBERSORT analysis also showed that the risk score exhibited apparent concordance with abundance of resting memory CD4 + T cell subsets and macrophages in M2 phase (Fig. 6D and supplementary Fig. 8D). These results were also validated by xCELL algorithm (supplementary Fig. 8G and 8 H). By investigating the association between biomarkers of different phenotypes of macrophages and the risk score in TCGA and CGGA cohorts, we found that cholesterol metabolic status was tightly correlated to tumor-associated macrophage (TAM) and macrophages in M2 (Fig. 6E and supplementary Fig. 8E) [44]. Additionally, GSVA analysis suggested that the risk signature was positively correlated with the T-helper 1/2 type cell-mediated immune response (GO:0042088 and GO: 0042092), T-helper 1/2 type cell production (GO:2,000,556 and GO: 2,000,553) and natural killer (NK) cell mediated cytotoxicity directed against tumor cell target (GO:0002860). However, we also found that there was no significant correlation between cholesterol metabolism and T cell mediated immune response to tumor cells (GO:0002842 and GO:0002852). These results suggested that enhanced cholesterol metabolism may serve as an inhibitor in T-cell immunity in LGGs. (Fig. 6F and supplementary Fig. 8F). In brief, cholesterol metabolism might be able to shape the tumor immunologic microenvironment by regulating the phenotypic polarization of T cells and macrophages.

### Prognostic validity of the cholesterol metabolism-related signature

**Fig. 7:**
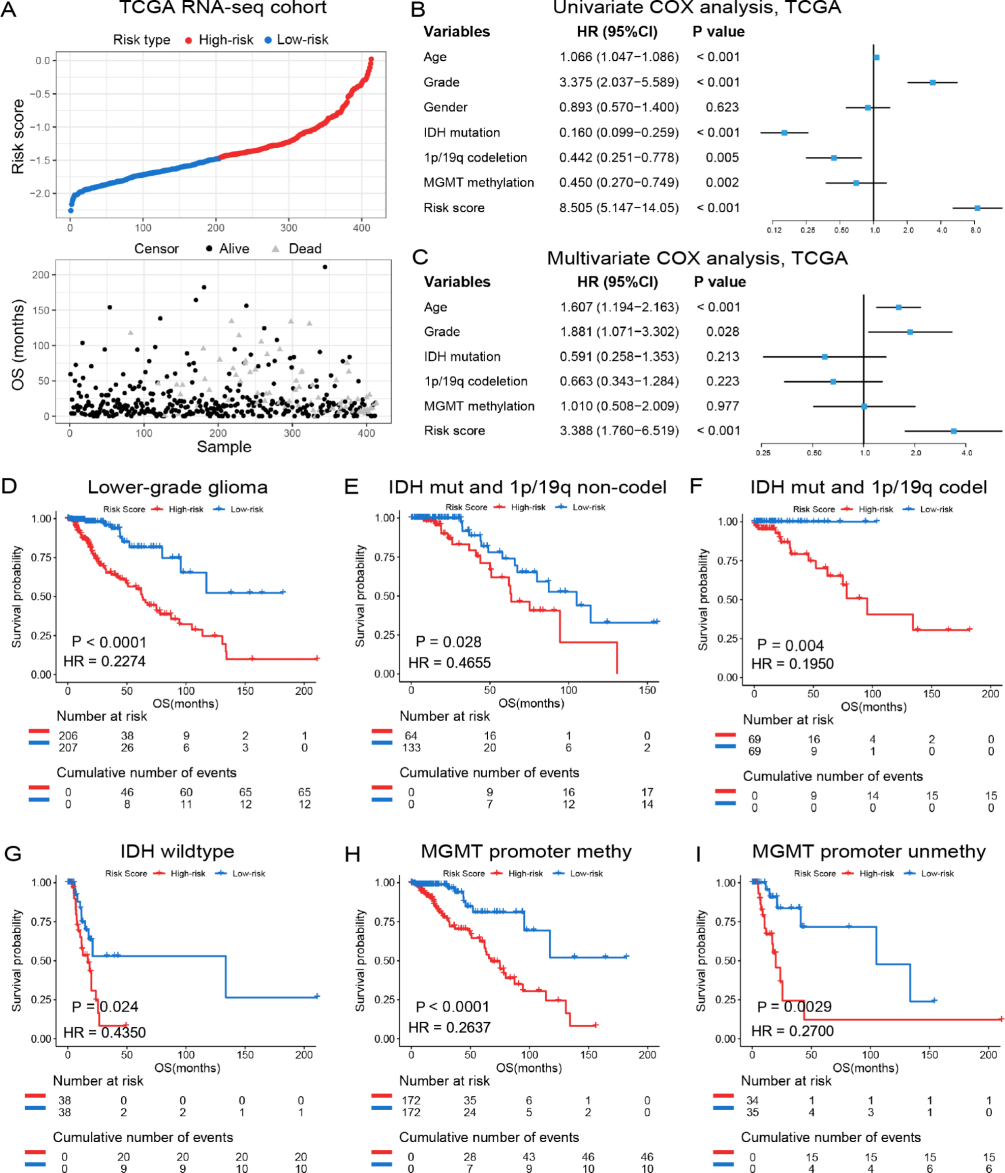
The prognostic value of the risk signature in TCGA cohort. **(A)** The distribution of the risk score and survival overview of LGG patients. **(B, C)** Univariate and multivariate Cox regression analyses of risk score and other clinicopathological features. **(D)** K-M survival analysis of the risk signature in LGG patients. **(E-G)** K-M survival analyses of the risk signature in LGG patients stratified by *IDH* mutation and 1p/19q codeletion status. **(H, I)** K-M survival analyses of the risk signature in LGG patients stratified by *MGMT* promoter methylation status

As we mentioned above, cholesterol metabolism might play an essential role in the malignant progression and tumor microenvironment remodeling. Therefore, we further assessed the impact of cholesterol metabolic status on the prognosis of patients with LGGs in TCGA and CGGA cohort. We first divided patients into low and high-risk group based on the median value of risk scores. The overview of living conditions and the risk score suggested that LGG patients with high or low risk score had different proportion of deaths in both TCGA (Fig. 7A, P < 0.0001) and CGGA (supplementary Fig. 9A, P < 0.0001) database. The univariate and multivariate Cox regression analyses showed that the risk signature was an independent prognostic factor for patients with LGG (Fig. 7B C, supplementary Fig. 9B and 9 C). We further performed survival analysis to detect the prognostic value of the risk signature in TCGA and CGGA cohort. The results revealed that high risk score significantly reduced overall survival (OS) time among LGG patients (P < 0.0001, Fig. 7D and supplementary Fig. 9D). Besides, LGG samples were divided into three groups comprising *IDH*-mutant LGG without 1p/19q codeletion, *IDH*-mutant LGG with 1p/19q codeletion, and *IDH*-wild type LGG. Subsequently, K-M survival analysis was performed to investigated the prognostic value of the risk signature in these three groups, separately. We found that the risk score was negatively correlated to the OS of patients in all three groups, except *IDH*-mutant with 1p/19q codeletion group in CGGA cohort (Fig. 7E and G and supplementary Fig. 9E-9G). Moreover, when stratified LGG patients by *MGMT* promoter methylation, high risk scores also tended to be associated with poor clinical outcomes (Fig. 7H and I, supplementary Fig. 9H and 9I). To further verify the prognostic value of the cholesterol metabolism-related risk signature, K-M survival analysis and Cox regression analysis were performed in LGG samples from GSE16011 dataset. The results also indicated that the risk signature was an independent prognostic factor of LGG patients (supplementary Fig. 9J and K) These findings indicated that the risk signature was an independent prognosticator in LGG.

### Construction of a nomogram model based on the risk signature

**Fig. 8:**
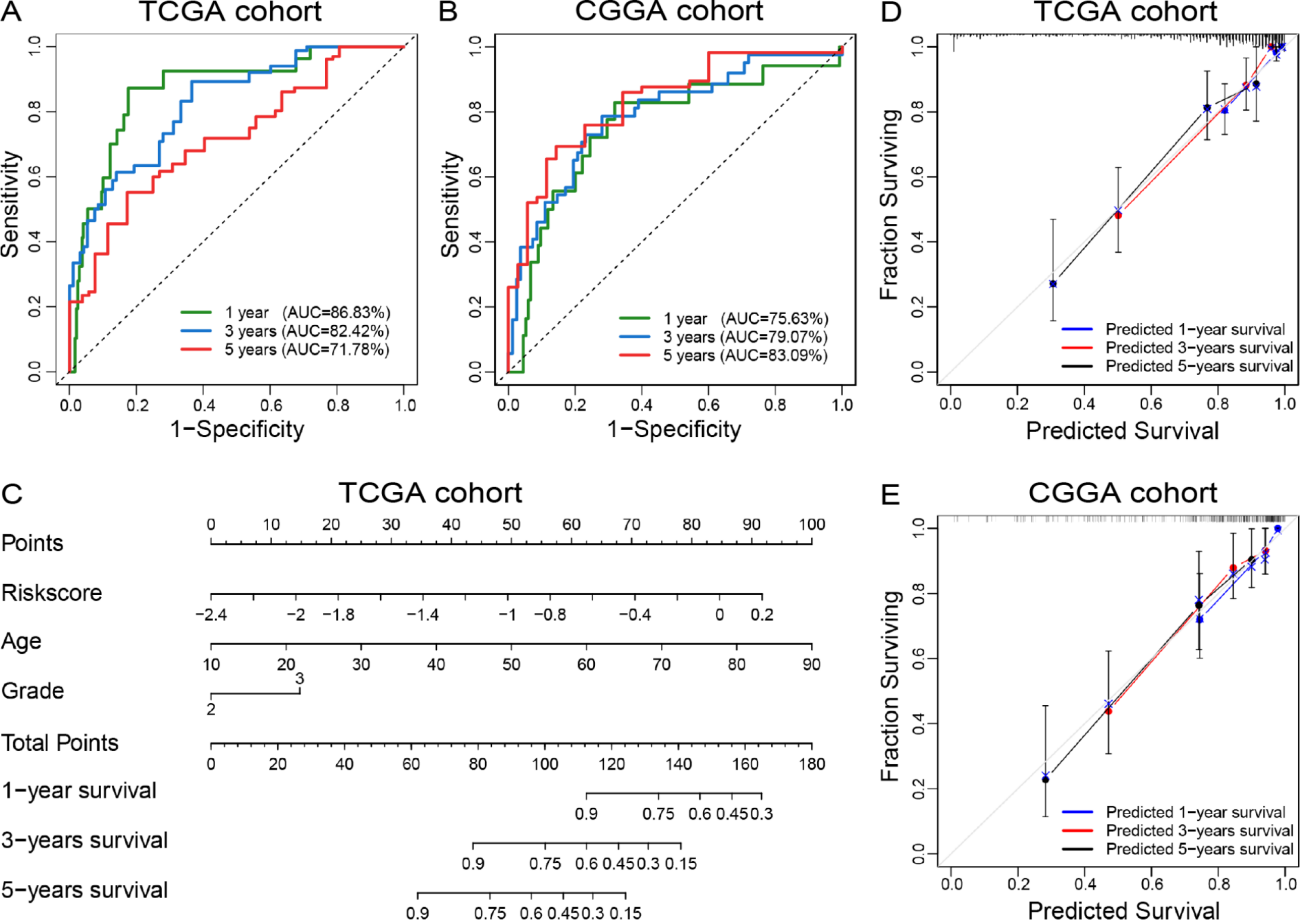
A nomogram model for predicting clinical outcome of LGG patients in TCGA and CGGA cohorts. **(A, B)** The ROC curve analyses were performed to predict 1-, 3-, and 5-year survival of LGG patients according to risk score. **(C)** A nomogram model was constructed by integrating the risk score with the clinicopathologic characteristics in the TCGA cohort. **(D, E)** Calibration curves of nomogram model for predicting 1-year (blue line), 3-year (red line), and 5-year (black line) survival of LGG patients

To improve the clinical application value of this cholesterol metabolism-related signature, ROC curve analysis was performed to assess the prediction accuracy of risk signature for the 1-, 3- and 5-year survival rate. We found that the risk signature showed superior predictive value, with high time-dependent AUC in TCGA (1 year: 86.83%, 3 years: 82.42%, 5 years: 71.78%) and CGGA (1 year: 75.63%; 3 years: 79.07%, 5 years: 83.09%) cohorts (Fig. 8A and B). The Schoenfeld individual test suggested that the risk of all variables did not change along with time in both TCGA and CGGA cohort (supplementary Fig. 10A and 10B). On this foundation, independent prognostic factors for overall survival time were selected to construct a nomogram model (Fig. 8C). The C-indices were 0.864 (95% CI: 0.272 ~ 1.728) and 0.809 (95% CI: 0.383 ~ 1.617) in TCGA and CGGA cohort, respectively. Besides, the calibration plot of survival rate also showed satisfactory concordance between training and validation cohorts (Fig. 8D and E). Thus, the risk signature could serve as an important reference for predicting the prognosis of LGG patients.

### ***SOAT1*** enhanced the proliferation and migration capability of glioma cells

**Fig. 9:**
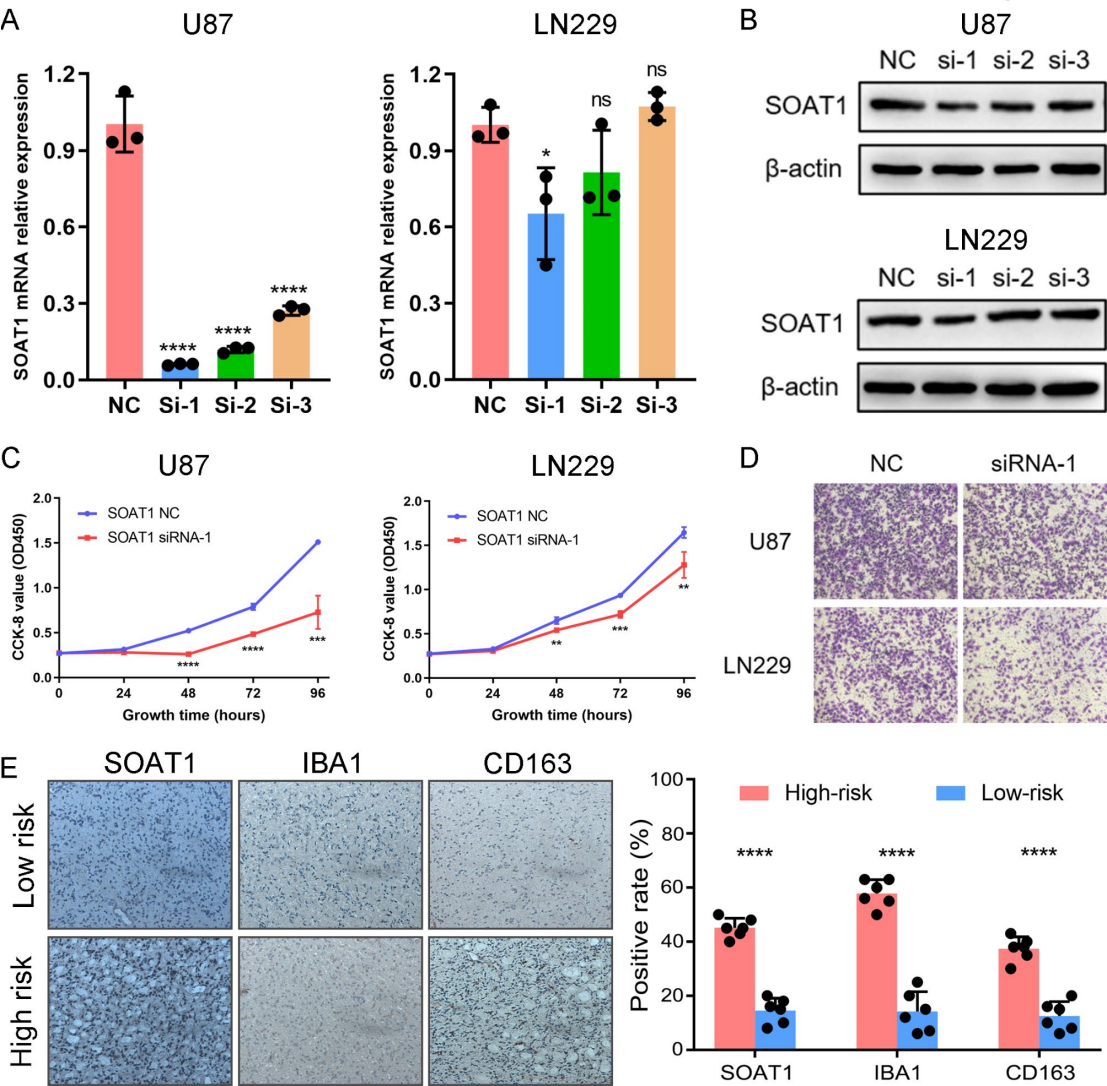
SOAT1 promotes proliferation and migration of glioma cells in vitro.**(A)** The qPCR assay of SOAT1 mRNA expression in U87 and LN229 cell lines after transfection of specific siRNA and negative control. **(B)** The western blot analysis of SOTA1 protein expression in U87 and LN229 cell lines after transfection of specific siRNA and negative control. **(C)** CCK-8 assay was performed to detect the proliferative capacity of U87 and LN229 cell lines. **(D)** Transwell migration assay was performed to detect the migration capacity of U87 and LN229 cell lines. **(E)** IHC staining and positive rate statistics of SOAT1, IBA1 and CD163 in LGG samples from the CGGA cohort

To further validate the results of bioinformatics analyses, in vitro experiments were performed with U87 and LN229 cell lines. First, the glioma cell lines were transfected with specific siRNA to knock down the mRNA expression of *SOAT1*, which was the main contributor of the risk signature. qPCR and Western Blot assay demonstrated that mRNA and protein expressions of *SOAT1* were significantly decreased in both U87 and LN229 cell lines post‑transfection with siRNA‑1 (Fig. 9A and B). Then, we performed CCK-8 and transwell migration assays to further evaluate the function of *SOAT1* in glioma cell lines. We found that knockdown of *SOAT1* could significantly suppressed the proliferation and migration capability of glioma cells (Fig. 9C and D). Besides, the IHC analysis showed that the expression level of SOAT1, IBA1 and CD163 were significantly increased in LGG samples from high-risk group, suggesting that cholesterol metabolism was positively correlated to the infiltration and M2 polarization of macrophages(Fig. 9E). These results indicated that cholesterol metabolism played a key role in malignant progression of gliomas [45, 46].

## Discussion

In recent years, increasing evidences have shown that metabolic reprogramming is one of the major hallmarks of malignant tumor [47, 48]. To meet the excessive energy consumption and biosynthetic demands associated with rapid proliferation, cancer cell generally exhibit metabolic disorders, involving glucose, amino acid, and lipid metabolism et al [49–51]. As a membrane constituent of mammalian cells, cholesterol plays an essential role in maintaining the integrity and fluidity of the membrane, which is also indispensable for the survival and proliferation of cancer cells [8, 9]. Metabolites of cholesterol, including bile acids, oxysterols, and steroid hormones can innate and promote a variety of human cancers [13, 15]. Besides, several studies have revealed that cholesterol accumulated in TME also participate in promoting cancer progression and suppressing immune responses. However, few studies focus on the significance of cholesterol metabolism in LGGs.

In this study, LGG samples from TCGA and CGGA databases were enrolled in consensus clustering analysis. The results showed that LGG samples could be divided into two clusters with different clinicopathological and prognostic characteristics based on the expression of cholesterol metabolism-related genes. To evaluate the cholesterol metabolic status and prognosis of LGG patients, an integrated cholesterol metabolism-related risk signature was constructed by least absolute shrinkage and selection operator (LASSO) Cox regression model. This signature could evaluate the functional roles and prognostic value of cholesterol metabolism in LGGs. By investigating the distribution of risk score stratified patients, we found that the risk score was positively correlated with malignant clinicopathologic characteristics. Besides, it is worth mentioning that 7p11.2 contains *EGFR*, while 10q23.3 contains the *PTEN* locus. Chr 7 amplification accompanied Chr 10 loss, which eventually leads to *EGFR* amplification and *PTEN* loss, was generally considered to be a representative genomic alteration in GBM [52, 53]. Therefore, the genomic alteration pattern of LGG samples in high-risk group was similar to GBM, suggesting that cholesterol metabolism might play a key role in the malignant progression of LGGs.

Bioinformatics analysis showed that the risk signature was tightly association with immune and inflammatory response, indicating a tight interaction between cholesterol metabolism and TIME in LGGs. In CIBERSORT analysis, the risk signature was positively correlated with the infiltration of resting memory CD4 + T cells and macrophages in M2 phase, which were considered to promote immunosuppression and malignant progression of tumors [54, 55]. Relevant studies pointed out that cancer cells could promote cholesterol efflux in TAMs and polarize TAMs towards an M2-like phenotype, which ultimately accelerates the malignant progression of cancers [56, 57]. Moreover, some research has shown that high levels of cholesterol activate the Toll-like receptor (TLR) present on macrophages and generate a chronic inflammation with in the TME that promotes cancer progression [58]. In this study, the IHC analysis also suggested that cholesterol metabolism was positively correlated to the infiltration and M2 polarization of macrophages. Extracellular cholesterol accumulation in the TME had been shown to induce T cell functional exhaustion by promoting several well-known immune checkpoints, such as *PD-1*, *TIM-3*, *2B4*, and *LAG-3* [19]. In addition, 22-hydroxycholesterol (22HC), a metabolite of cholesterol, could inhibit T cell-mediated anti-tumor immunity by activating liver X receptors (LXRs) signaling pathway [59]. More importantly, cholesterol metabolism could facilitate CD4 + and CD8 + T-cell differentiation and acquisition of effector function [60, 61]. In the present study, the GSVA analysis also showed that enhanced cholesterol metabolism played an inhibitory role in T cell-mediated antitumor immune response in gliomas. Beyond TAMs and T cells, 22HC, 24-hydroxycholesterol (24HC) and 27-hydroxycholesterol (27HC) could also recruit neutrophils, which are emerging as an important immunosuppressive population in the TME [62–64]. In brief, cholesterol metabolic status had significant impact on TIME and this finding provided an important reference for further improve the efficacy of immunotherapy of LGGs.

To investigate the prognostic value of this risk signature, we performed K-M survival and COX regression analyses in both TCGA and CGGA cohorts. Even if we divided patients into different groups according to molecular pathological characteristics, the risk signature still showed high prognostic value in each group. For *IDH* mutant patients with 1p/19q codeletion in CGGA cohort, the K-M survival analysis showed no statistical difference, which was mainly due to the small number of samples and short follow-up time. In brief, the results suggested that the risk signature was an independent prognostic factor for LGG patients. In this risk signature, each single gene was key mediator in the cholesterol metabolic signaling pathways and could serve as a prognostic factor of LGG patients. However, by constructing a risk signature, we could estimate the cholesterol metabolic status and predict prognosis of LGG patients more precisely. To improve the clinical application value of the risk signature, we also developed a nomogram model to predict 1-, 3- and 5-year survival of LGG patients. Compared with other LGG prognostic models proposed by previous studies [65, 66], the cholesterol metabolism-related signature showed similar prognostic value while accurately assessing cholesterol metabolism of gliomas. Therefore, the cholesterol metabolism-related signature was a reliable and robust prognostic indicator.

Because of the vital functions of cholesterol metabolism in cancer imitation and progression, impeding active cholesterol metabolism became a feasible treatment strategy and caused extensive concern [16]. For example, HMG-CoA reductase (*HMGCR*) inhibitor, which was the most wildly used anti-cholesterol drug, has been proved to decrease mortality and prolong survival of patients with colorectal or prostate cancer [67–69]. In addition, inhibitors of Acyl Coenzyme A: Cholesterol Acyltransferases 1 (*ACAT1*), which also known as sterol O-acyltransferase 1 (*SOAT1*), could suppress tumor growth by downregulating cholesterol esterification (CE) in leukemia and triple-negative breast cancer [45, 70]. In the present study, as the most important contributor of the risk signature (Regression coefficients = 0.2773), the biological function of *SOAT1* were also investigated in glioma cell lines. The results showed that silencing *SOAT1* expression suppressed the proliferation and migration capacity of glioma cells, which may provide novel target for the treatment of gliomas. Interestingly, both agonists and inverse agonists of LXR have shown significant efficacy for the treatment of multiple human cancers, mainly by inhibiting cell proliferation and regulating anti-tumor immune responses [71–73]. Based on findings of our study, we hypothesized that the eight genes in the cholesterol metabolism-related signature might also serve as promising therapeutic targets of LGGs. However, these conjectures need more experimental verification.

In brief, through comprehensively analyzing clinical and transcriptomic profiling data in TCGA and CGGA cohorts, we identified a cholesterol metabolism-related signature to evaluate the cholesterol metabolic status and predict the prognosis of patients with LGGs. Moreover, we found that cholesterol was involved in macrophage- and T cell-mediated antitumor immunity. Finally, the risk signature was revealed to be an independent prognostic indicator of LGG patients. This study was the first to characterize cholesterol metabolism in LGGs clinically and molecularly. Although, more in-depth exploration and prospective cohort studies were needed to estimate the significances of cholesterol metabolism in LGGs. These findings shed light on functional roles of cholesterol metabolism in LGGs and provided promising targets to enhance the anti-tumor therapies.

## Electronic supplementary material

Below is the link to the electronic supplementary material.

Supplementary Material 1

Supplementary Material 2

Supplementary Material 3

## Data Availability

The data that support the findings of this study are openly available in TCGA dataset at https://portal.gdc.cancer.gov/projects/TCGA-LGG, CGGA dataset at http://www.cgga.org.cn/download.jsp, and Ivy cohort at http://glioblastoma.alleninstitute.org/static/download.html.
