# Supplementary figures and images for: Comprehensive analysis of the clinical and biological significances of cholesterol metabolism in lower-grade gliomas
Source: BMC Cancer. 2023 Jul 24;23:692. doi: 10.1186/s12885-023-10897-0 (PMC10364387; doi:10.1186/s12885-023-10897-0)

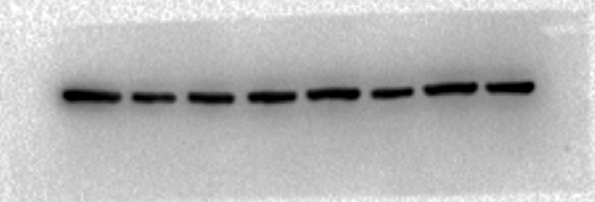

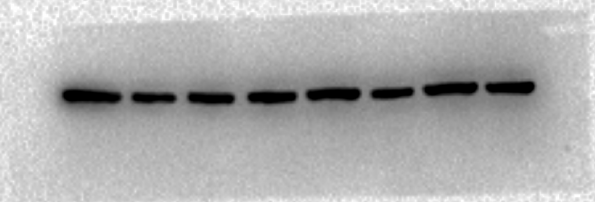

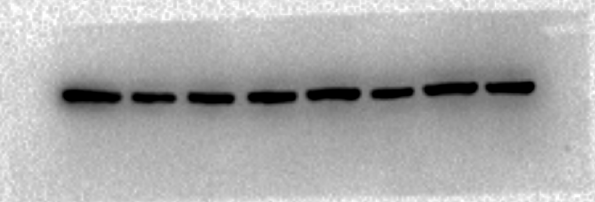

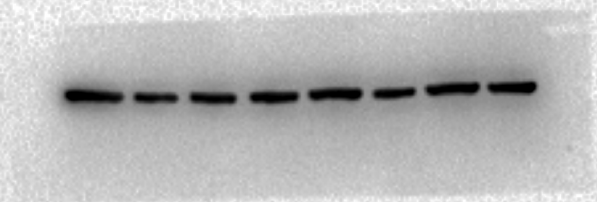

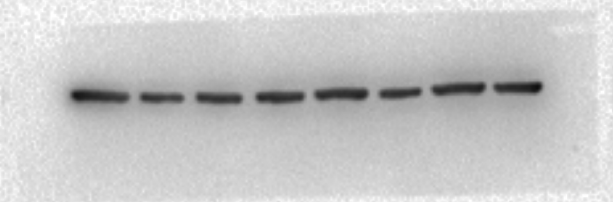

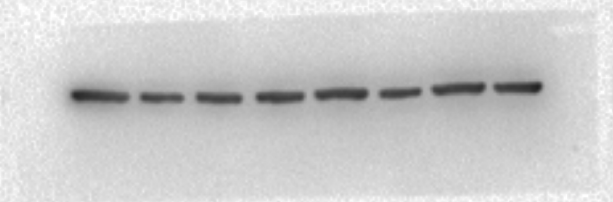

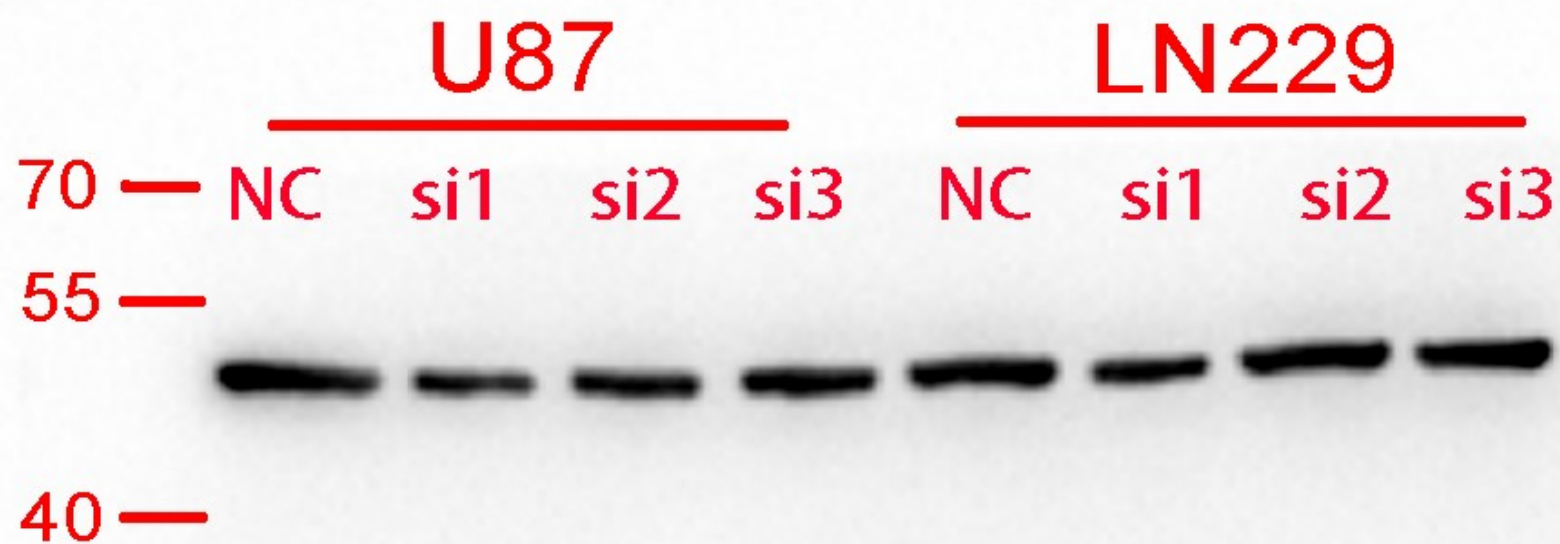

SOAT1

Supplement: Supplementary file 1 — Supplementary Material 1 [file 12885_2023_10897_MOESM1_ESM.pdf]

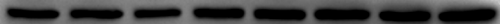

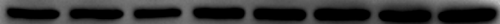

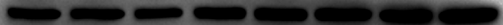

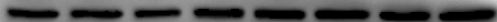

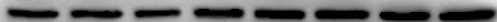

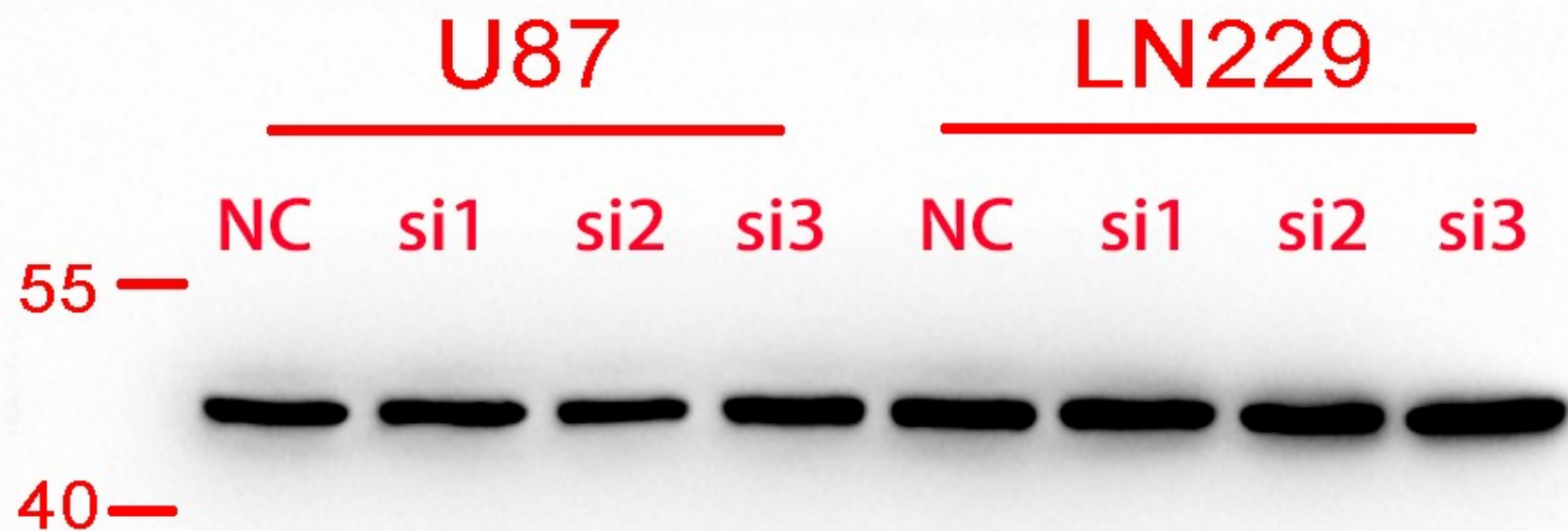

β-actin

Supplement: Supplementary file 2 — Supplementary Material 2 [file 12885_2023_10897_MOESM2_ESM.pdf]
