# Supplementary material for: Comprehensive analysis of the clinical and biological significances of cholesterol metabolism in lower-grade gliomas
Source: BMC Cancer. 2023 Jul 24;23:692. doi: 10.1186/s12885-023-10897-0 (PMC10364387; doi:10.1186/s12885-023-10897-0)
Supplement: Supplementary file 3 — Supplementary Material 3 [file 12885_2023_10897_MOESM3_ESM.docx]

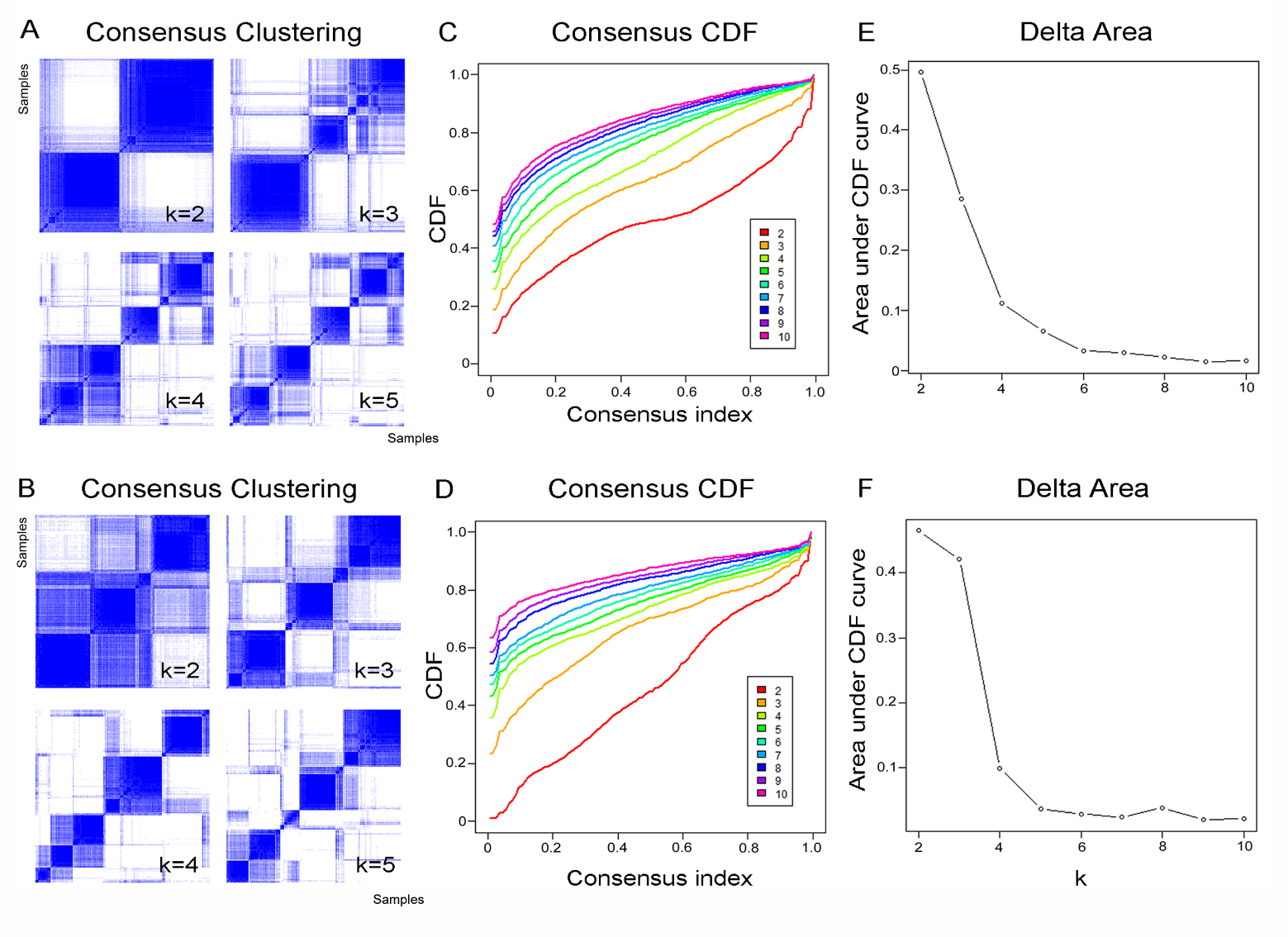


**Supplementary Figure 1.** Consensus clustering for cholesterol metabolism-related genes in LGG samples. The x and y axis represented to tumor samples of patients enrolled in this study. The bluer the area was in the figure, the greater probability the corresponding samples were grouped as one cluster(A, B). Consensus clustering matrix of LGG samples for k = 2 to k=5, respectively(C, D). Consensus clustering CDF for k = 2 to k = 10 (E, F). Relative change in area under CDF curve for k = 2 to k = 10.


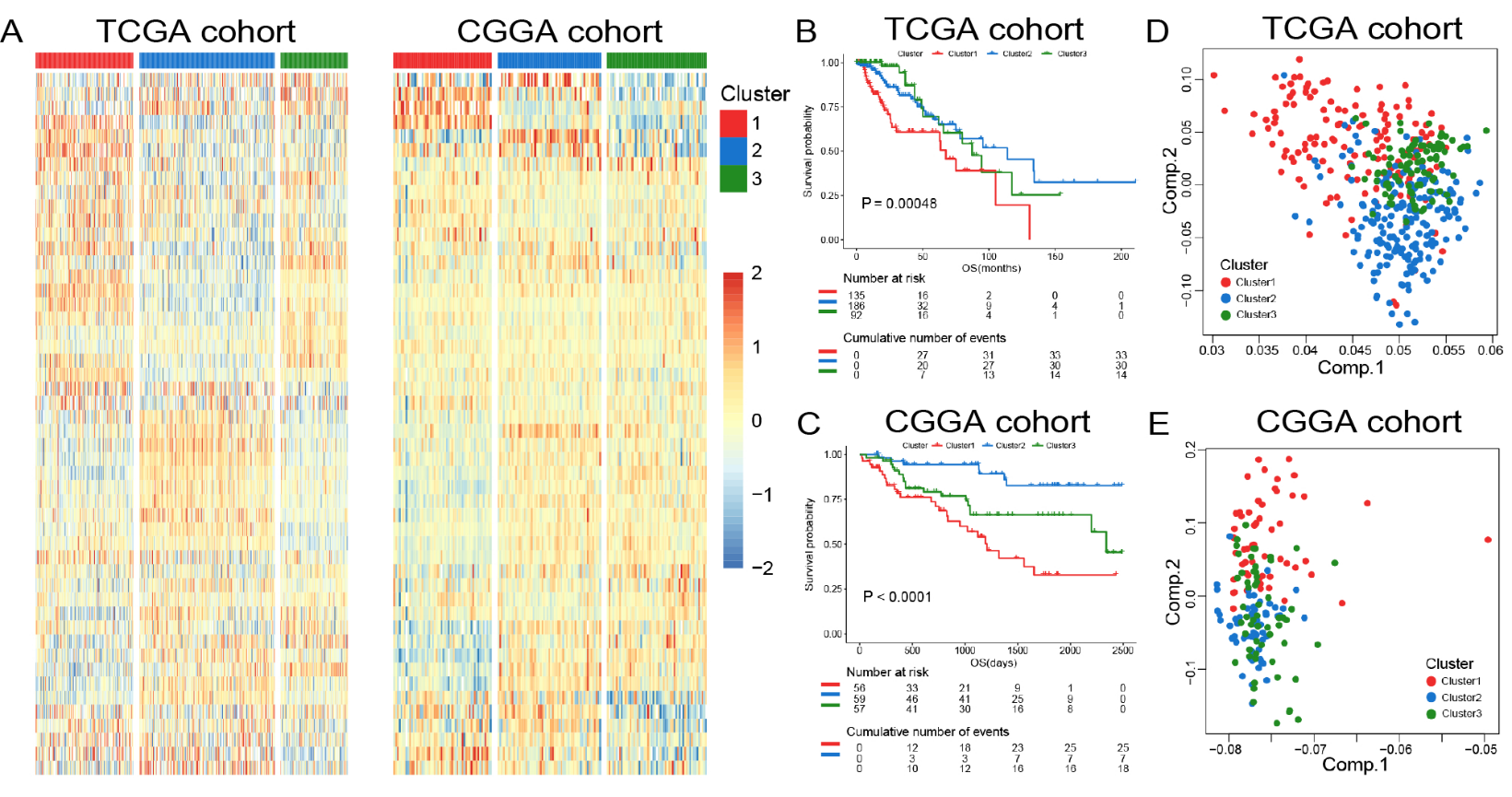


**Supplementary Figure 2.** Consensus clustering identified three cholesterol metabolism-related clusters in TCGA and CGGA cohorts.(A) Heatmap of three clusters identified by the cholesterol metabolism-related genes with high MAD (top 50). (B, C) K-M survival analyses of patients in three clusters. (D, E) Principal components analysis of three clusters.


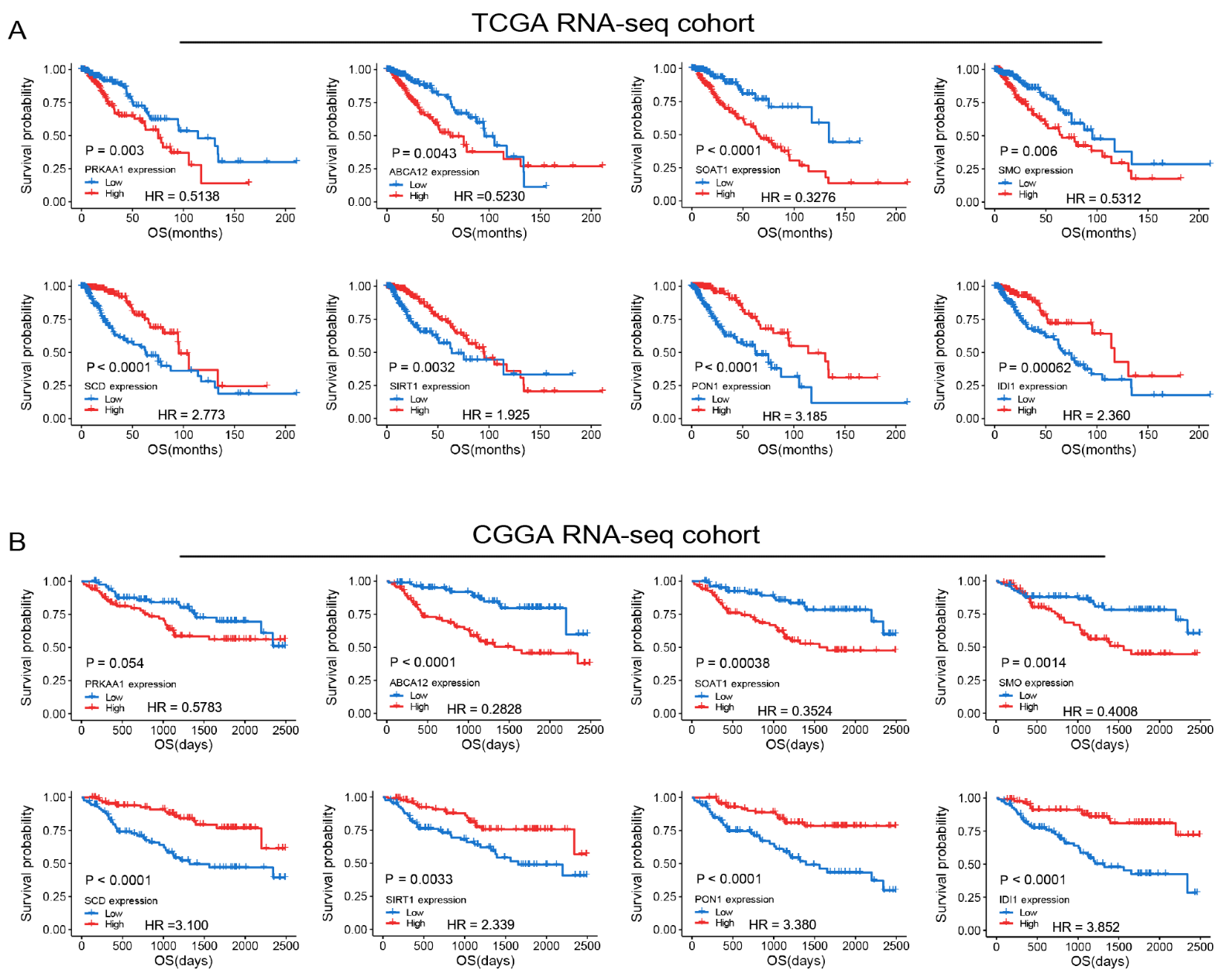


**Supplementary Figure 3.** K-M survival analysis of each gene in the cholesterol metabolism-related signature in TCGA (A) and CGGA cohorts (B).


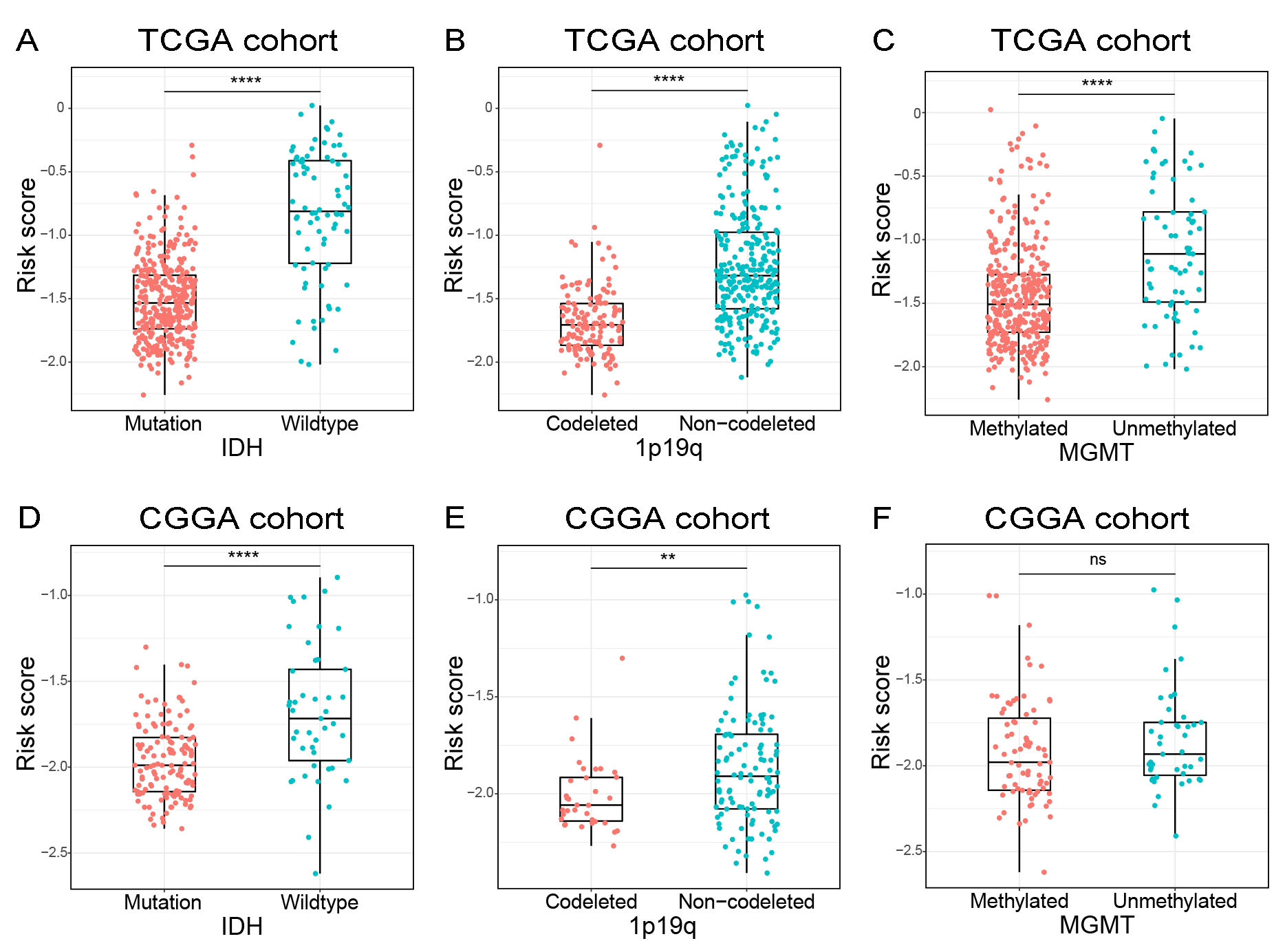


**Supplementary Figure 4.** The association between risk score and *IDH* mutation (A, D), 1p/19q codeletion (B, E) and *MGMT* promoter methylation status (C, F) of LGG patients in TCGA and CGGA cohorts. ** P <0.01, **** P <0.0001, ns: no statistical significance.


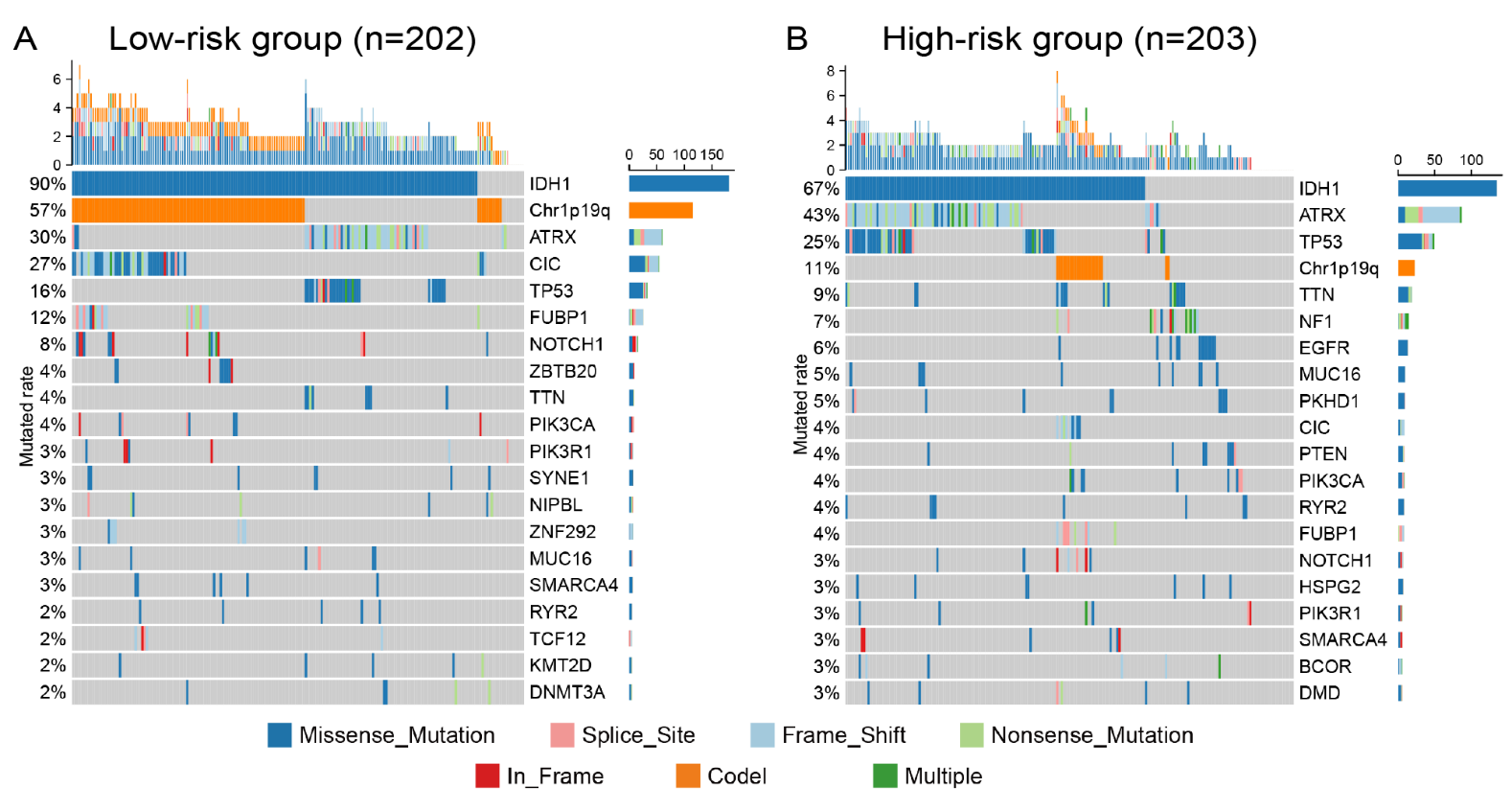


**Supplementary Figure 5.** Comparison of somatic mutations between LGG samples in low-risk (A) and high-risk group (B).


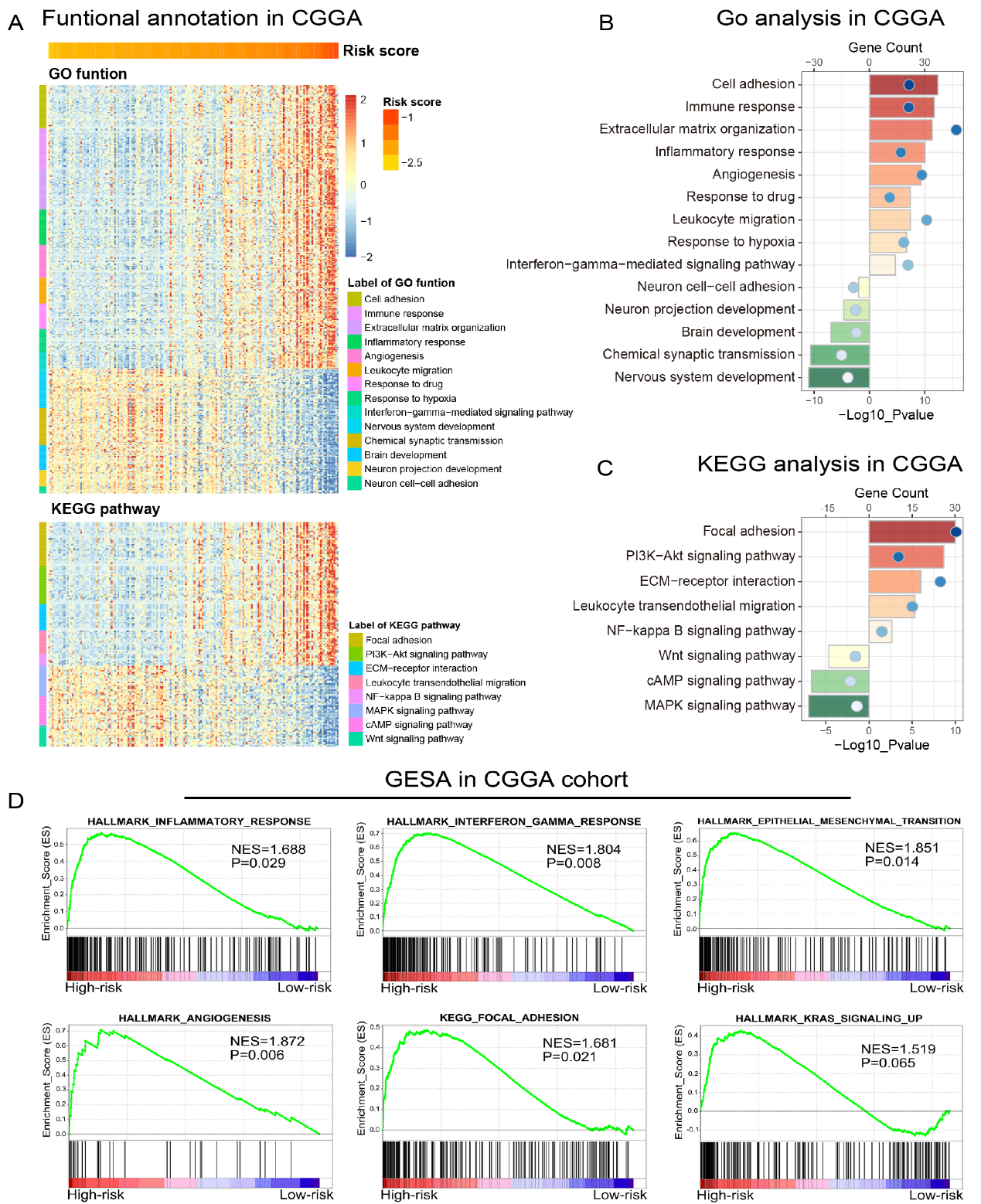


**Supplementary Figure 6.** Functional annotation of the risk signature in CGGA cohort. (A) The heatmaps showed the association between risk score and cholesterol metabolism-related biological functions and pathways, which were identified by GO and KEGG pathway analyses. (B, C) GO and KEGG pathway analyses were performed via the DAVID website to investigate the biological processes tightly correlated to the risk signature. (D) GSEA analysis was performed to explore the biological functions that were significantly enriched in LGG samples from high-risk group.


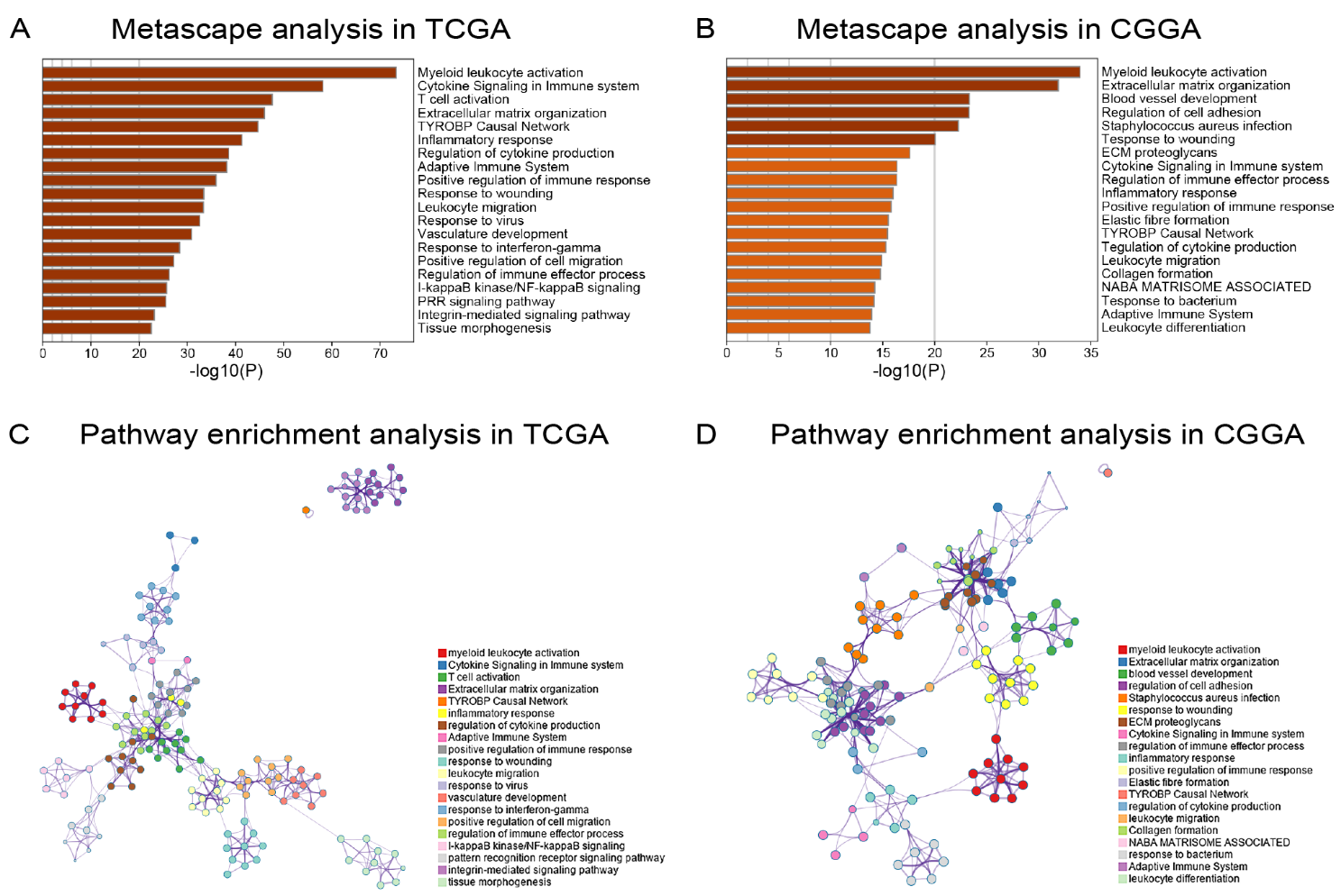


**Supplementary Figure 7.** Bar graph (A, B) and network (C, D) of enriched functions and pathways which were identified by Metascape with cholesterol metabolism-related genes in TCGA and CGGA cohorts. In the network, the nodes colored by their cluster ID.


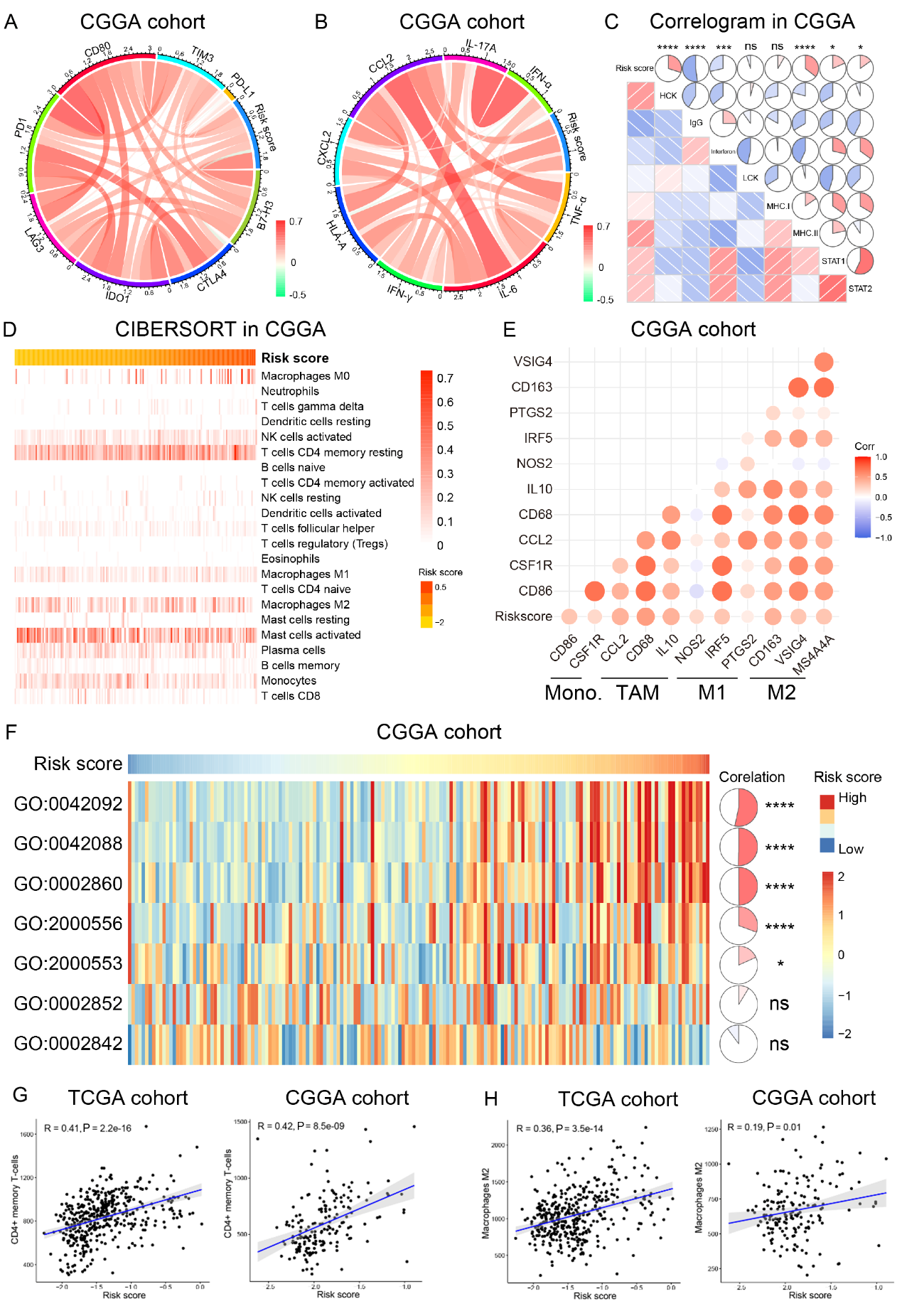


**Supplementary Figure 8.** Cholesterol metabolism participated in the regulation of tumor immune microenvironment in CGGA cohort**.** (A) Correlation analysis between the risk signature and immune checkpoints in LGG samples. (B) Correlation analysis between the risk signature and inflammatory hallmarks in LGG samples. (C) The association between the risk signature and inflammatory metagenes. (D) The proportion of immune cells infiltrated into LGG samples was evaluated by CIBERSORT. (E) The association between the risk signature and biomarkers of monocytes and macrophages in different phase. (F) The relationship between the risk signature and T-cell-related immunity. GO:0042092: T-helper 2 type immune response; GO:0042088: T-helper 1 type immune response; GO:0002860: positive regulation of natural killer cell-mediated cytotoxicity directed against tumor cell target; GO:2000556: positive regulation of T-helper 1 cell cytokine production; GO:2000553: positive regulation of T-helper 2 cell cytokine production; GO:0002842: positive regulation of T cell-mediated immune response to tumor cell; GO:0002852: regulation of T cell-mediated cytotoxicity directed against tumor cell target. (G, H) The association of risk signature with CD4+ memory T cells and macrophages M2 in LGG samples was evaluated by xCELL algorithm. * P <0.05, *** P <0.001, **** P<0.0001, ns: no statistically significant.


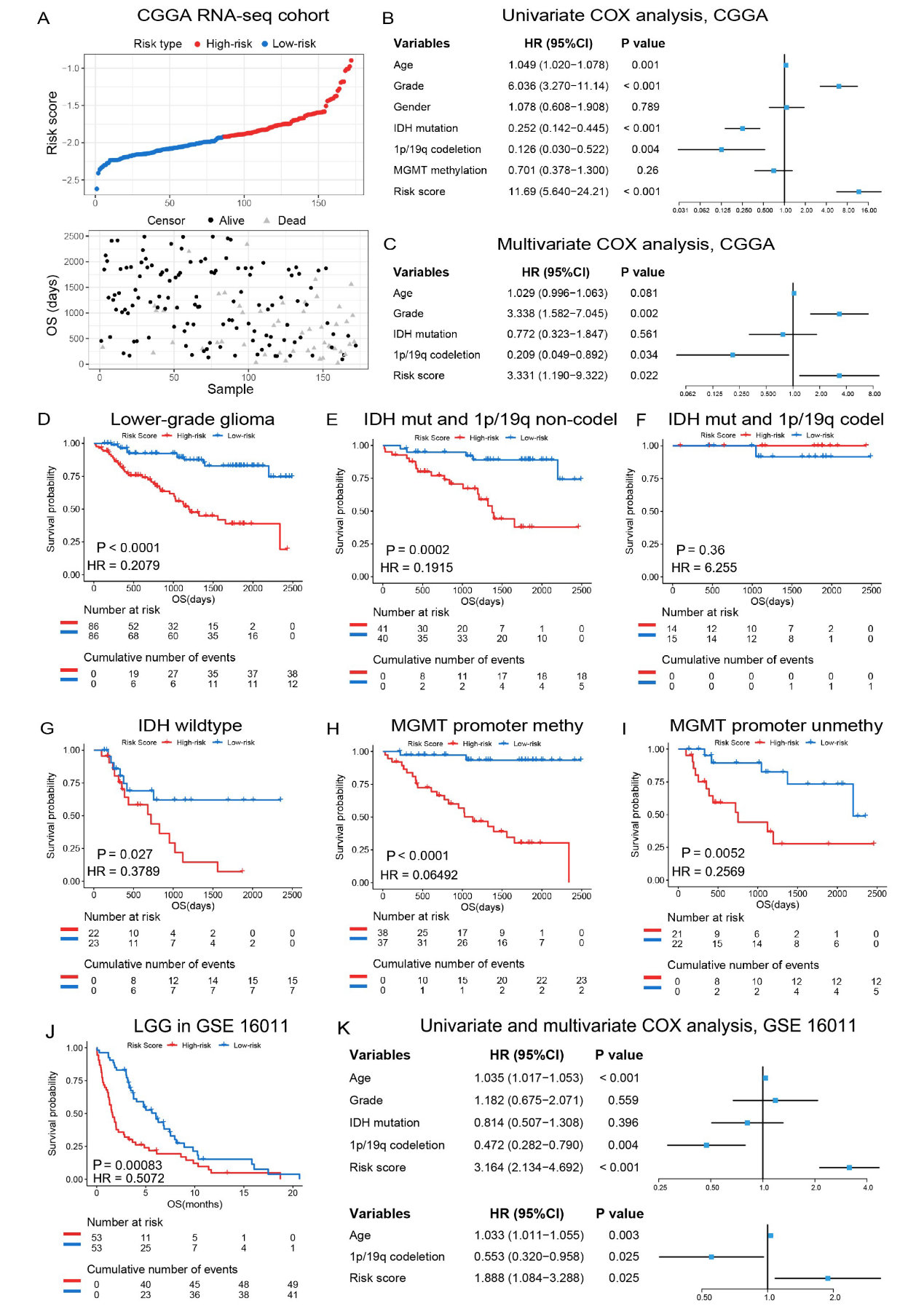


**Supplementary Figure 9.** The prognostic value of the risk signature in CGGA and GSE16011 cohorts. (A) The distribution of the risk score and survival overview of LGG patients in CGGA cohort. (B, C) Univariate and multivariate Cox regression analyses of risk score and other clinicopathological features in CGGA cohort. (D) K-M survival analysis of the risk signature in LGG patients in CGGA cohort. (E-G) K-M survival analyses of the risk signature in LGG patients stratified by *IDH* mutation and 1p/19q codeletion status in CGGA cohort. (H, I) K-M survival analyses of the risk signature in LGG patients stratified by *MGMT* promoter methylation status in CGGA cohort. (J) K-M survival analysis of the risk signature in LGG patients in GSE16011 cohort. (K) Univariate and multivariate Cox regression analyses of risk score and other clinicopathological features in GSE16011 cohort.


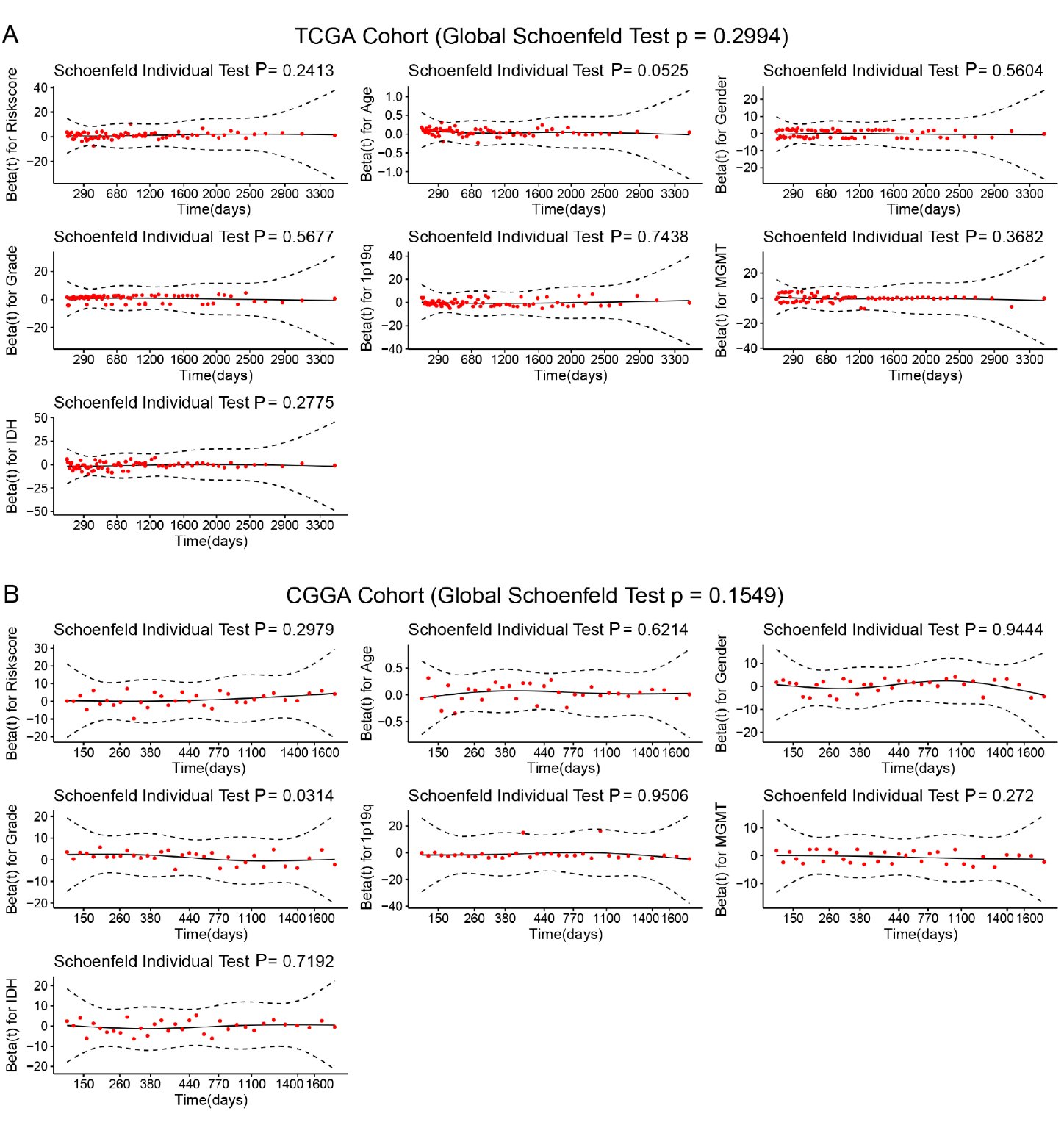


**Supplementary Figure 10.** Schoenfeld individual test. (A) Schoenfeld individual test of all variables in TCGA cohorts. (B) Schoenfeld individual test of all variables in CGGA cohorts.

**Supplementary Table 1**

**Cholesterol metabolism-related gene list**

| AACS | EHD1 | LDLRAP1 | PON1 |
| --- | --- | --- | --- |
| ABCA1 | ELOVL6 | LPL | POR |
| ABCA12 | EPHX2 | LRP1 | PPARA |
| ABCA5 | ERLIN1 | LRP6 | PPARD |
| ABCG1 | ERLIN2 | LRP8 | PPARG |
| ABCG4 | FASN | LSS | PRKAA1 |
| ACACA | FDFT1 | MBTPS1 | PTCH1 |
| ACACB | FDPS | MLC1 | RAN |
| ACADL | FGF1 | MSR1 | SCAP |
| ACADVL | GGPS1 | MVD | SCARB1 |
| APOC1 | GNB3 | MVK | SCD |
| APOC2 | GPAM | NFE2L1 | SEC14L2 |
| APOE | GPLD1 | NFKBIA | SIRT1 |
| APOM | GPS2 | NFYB | SMO |
| ARV1 | GRAMD1C | NFYC | SOAT1 |
| CD36 | HMGCR | NPC1 | SOD1 |
| CETP | HMGCS1 | NPC2 | SP1 |
| CYP51A1 | IDI1 | NR1H2 | SQLE |
| DGAT2 | INHBA | NR1H3 | SREBF1 |
| DHCR7 | KPNB1 | NR1H4 | SREBF2 |
| EGF | LDLR | PLTP | STX12 |
| AACS | EHD1 | LDLRAP1 | PON1 |

**Supplementary Table 1.** Cholesterol metabolism-related gene list.

| **Supplementary Table 2**  **Characteristics of patients in cluster 1 and cluster 2 in TCGA cohort** | | | | | | | |
| --- | --- | --- | --- | --- | --- | --- | --- |
| **Characteristic** | | **N** | **Cluster 1** | **Cluster 2** | | | **p value** |
| **Total cases** | | 413(100%) | 224(54.2%) | | 189(45.8%) | |  |
| **Gender** | |  |  |  | | 0.1407 | |
|  | Male | 226(54.7%) | 130(31.5%) | 96(23.2%) | |  | |
|  | Female | 187(45.3%) | 94(22.8%) | 93(22.5%) | |  | |
| **Age (years)** | |  |  |  | | 0.9973 | |
|  | ≤40 | 201(48.7%) | 109(26.4%) | 92(22.3%) | |  | |
|  | ＞40 | 212(51.3%) | 115(27.8%) | 97(23.5%) | |  | |
| **Grade** | |  |  |  | | 0.0802 | |
|  | 2 | 197(47.7%) | 98(23.7%) | 99(24.0%) | |  | |
|  | 3 | 216(52.3%) | 126(30.5%) | 90(21.8%) | |  | |
| **Histological type** | |  |  |  | | <0.0001 | |
|  | astrocytoma | 149(36.1%) | 118(28.6%) | 31(7.5%) | |  | |
|  | oligoastrocytoma | 108(26.2%) | 66(16.0%) | 42(10.2%) | |  | |
|  | oligodendroglioma | 156(37.8%) | 40(9.7%) | 116(28.1%) | |  | |
| **Subtype*** | |  |  |  | | <0.0001 | |
|  | Classical | 34(8.2%) | 32(7.7%) | 2(0.5%) | |  | |
|  | Mesenchymal | 26(6.3%) | 24(5.8%) | 2(0.5%) | |  | |
|  | Proneural | 197(47.7%) | 90(21.8%) | 107(25.9%) | |  | |
|  | Neural | 95(23.0%) | 23(5.6%) | 72(17.4%) | |  | |
| **IDH Status*** | |  |  |  | | <0.0001 | |
|  | Mutation | 335(81.1%) | 164(39.7%) | 171(41.4%) | |  | |
|  | Wildtype | 76(18.4%) | 59(14.3%) | 17(4.1%) | |  | |
| **MGMT Promoter** | |  |  |  | | 0.0009 | |
|  | Methylation | 344(83.3%) | 174(42.1%) | 170(41.2%) | |  | |
|  | Unmethylation | 69(16.7%) | 50(12.1%) | 19(4.6%) | |  | |
| **1p/19q** | |  |  |  | | <0.0001 | |
|  | Codel | 138(33.4%) | 15(3.6%) | 123(29.8%) | |  | |
|  | Intact | 275(66.6%) | 209(50.6%) | 66(16.0%) | |  | |

***** meant part of the data was missing. The percentage of each part did not add up to 100% because the decimal portion was rounded.

**Supplementary Table 2.** Characteristics of patients in cluster 1 and cluster 2 in TCGA cohort.

| **Supplementary Table 3**  **Characteristics of patients in cluster 1 and cluster 2 in CGGA cohort** | | | | | |
| --- | --- | --- | --- | --- | --- |
| **Characteristic** | | **N** | **Cluster 1** | **Cluster 2** | **p value** |
| **Total cases** | | 172(100%) | 55(32.0%) | 117(68.0%) |  |
| **Gender** | |  |  |  | 0.8868 |
|  | Male | 105(61.0%) | 34(19.8%) | 71(41.3%) |  |
|  | Female | 67(39.0%) | 21(12.2%) | 46(26.7%) |  |
| **Age (years)** | |  |  |  | 0.5060 |
|  | ≤40 | 97(56.4%) | 29(16.9%) | 68(39.5%) |  |
|  | ＞40 | 75(43.6%) | 26(15.1%) | 49(28.5%) |  |
| **Grade** | |  |  |  | 0.0013 |
|  | 2 | 105(61.0%) | 24(14.0%) | 81(47.1%) |  |
|  | 3 | 67(39.0%) | 31(18.0%) | 36(20.9%) |  |
| **Histological type** | |  |  |  | <0.0001 |
|  | astrocytoma | 66(38.4%) | 36(21.0%) | 30(17.4%) |  |
|  | oligoastrocytoma | 67(39.0%) | 15(8.7%) | 52(30.3%) |  |
|  | oligodendroglioma | 39(22.6%) | 4(2.3%) | 35(20.3%) |  |
| **Subtype** | |  |  |  | <0.0001 |
|  | Classical | 23(13.4%) | 17(9.9%) | 6(3.5%) |  |
|  | Mesenchymal | 15(8.7%) | 15(8.7%) | 0(0.0%) |  |
|  | Proneural | 69(40.1%) | 13(7.6%) | 56(32.6%) |  |
|  | Neural | 65(37.8%) | 10(5.8%) | 55(32.0%) |  |
| **IDH Status** | |  |  |  | <0.0001 |
|  | Mutation | 127(73.8%) | 29(16.9%) | 98(57.0%) |  |
|  | Wildtype | 45(26.2%) | 26(15.1%) | 19(11.0%) |  |
| **MGMT Promoter*** | |  |  |  | 0.1778 |
|  | Methylation | 75(43.6%) | 16(9.3%) | 59(34.3%) |  |
|  | Unmethylation | 43(25.0%) | 14(8.1%) | 29(16.9%) |  |
| **1p/19q*** | |  |  |  | 0.0502 |
|  | Codel | 34(19.8%) | 6(3.5%) | 28(16.3%) |  |
|  | Intact | 110(64.0%) | 39(22.7%) | 71(41.3%) |  |

***** meant part of the data was missing. The percentage of each part did not add up to 100% because the decimal portion was rounded.

**Supplementary Table 3.** Characteristics of patients in cluster 1 and cluster 2 in CGGA cohort.
